# Analysis of a dynamic model of guard cell signaling reveals the stability of signal propagation

**DOI:** 10.1186/s12918-016-0327-7

**Published:** 2016-08-19

**Authors:** Xiao Gan, Réka Albert

**Affiliations:** Department of Physics, The Pennsylvania State University, University Park, PA USA

**Keywords:** Network model, Discrete dynamic model, Biological network, Signal transduction, Plant signaling, Attractor, Stomatal opening, Network reduction, Boolean conversion, Stable motif

## Abstract

**Background:**

Analyzing the long-term behaviors (attractors) of dynamic models of biological systems can provide valuable insight into biological phenotypes and their stability. In this paper we identify the allowed long-term behaviors of a multi-level, 70-node dynamic model of the stomatal opening process in plants.

**Results:**

We start by reducing the model’s huge state space. We first reduce unregulated nodes and simple mediator nodes, then simplify the regulatory functions of selected nodes while keeping the model consistent with experimental observations. We perform attractor analysis on the resulting 32-node reduced model by two methods: 1. converting it into a Boolean model, then applying two attractor-finding algorithms; 2. theoretical analysis of the regulatory functions. We further demonstrate the robustness of signal propagation by showing that a large percentage of single-node knockouts does not affect the stomatal opening level.

**Conclusions:**

Combining both methods with analysis of perturbation scenarios, we conclude that all nodes except two in the reduced model have a single attractor; and only two nodes can admit oscillations. The multistability or oscillations of these four nodes do not affect the stomatal opening level in any situation. This conclusion applies to the original model as well in all the biologically meaningful cases. In addition, the stomatal opening level is resilient against single-node knockouts. Thus, we conclude that the complex structure of this signal transduction network provides multiple information propagation pathways while not allowing extensive multistability or oscillations, resulting in robust signal propagation. Our innovative combination of methods offers a promising way to analyze multi-level models.

**Electronic supplementary material:**

The online version of this article (doi:10.1186/s12918-016-0327-7) contains supplementary material, which is available to authorized users.

## Background

Modeling offers a comprehensive way to understand biological processes by integrating the components involved in them and the interactions between components. Models can recapitulate and explain the emergent outcome(s) of the process [1, 2]. Representing cellular processes that involve many proteins and small molecules by a signal transduction network can reveal indirect relationships between components and provide new insight [3–5]. Such network usually consists of nodes representing biological entities, and edges representing interactions. Once a network has been constructed, dynamic modeling, where each node in the network is associated with a variable representing its abundance or activity, can further describe the behavior of the network. Dynamic models can have continuous variables whose change is described by differential equations [6], discrete variables described by discrete (logical) regulatory functions [7, 8], or a combination of continuous and discrete variables [9]. The major advantage of discrete dynamic and continuous-discrete hybrid models is that they use many fewer parameters than continuous models and thus need less parameter estimation [10–12]. Modeling allows one to analyze the biological system represented by the network *in silico*, when performing the relevant experiment is infeasible. It also helps identify general principles of biological systems [13, 14].

The biological process of stomatal opening in plants is a good example of a complex system wherein modeling leads to significant gain in understanding [15, 16]. Stomata are pores on leaf surfaces that allow the plant to exchange carbon dioxide (CO_2_) and oxygen with the atmosphere. Stomata are formed by two guard cells that can change shape: swelling of guard cells leads to stomatal opening; their shrinking leads to stomatal closure. The shape of each guard cell is directly controlled by water flow through the membrane, which is in turn controlled by ion flow. Different signals can affect the guard cell, changing its ion concentration in direct and indirect ways, resulting in stomatal opening or closure [17–19]. These signals include light of different wavelengths, CO_2_ concentration in the air, and plant hormones like abscisic acid (ABA). The regulation of stomatal opening is essential to plants, as it controls vital activities like the uptake of CO_2_ for photosynthesis, and the unavoidable water loss through evaporation [20]. Through extensive experimentation over several decades, more than 70 proteins and small molecules have been identified to participate in this process.

**Fig. 1:**
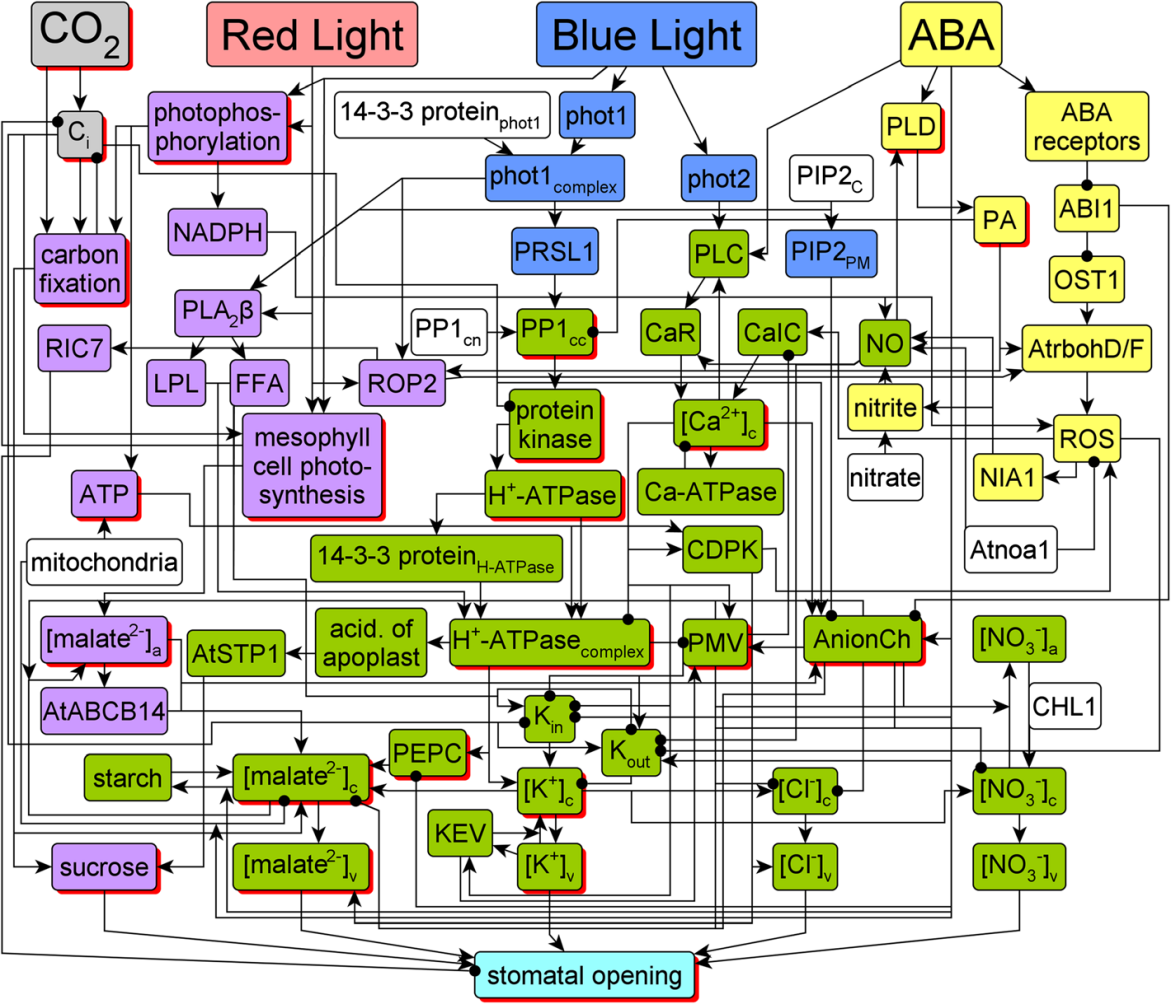
The signal transduction network responsible for stomatal opening, as reconstructed by Sun et al. [15]. The color of a node marks which signal regulates this node. Red nodes are regulated solely by red light. Blue nodes are regulated solely by blue light. Yellow nodes are regulated solely by ABA. Grey nodes are regulated by CO_2_. Purple nodes are regulated by both blue and red light. Green nodes are regulated by blue (and potentially, red) light and ABA. White nodes are source nodes not regulated by any of the four signals. To improve visualization, certain pairs of edges with the same starting or end nodes overlap. Nodes with multiple levels in the dynamic model are represented by red shadows; the others are Boolean. The full names of the network components denoted by abbreviated node names are given in Table 1. This figure and part of its caption is reproduced from Sun Z, Jin X, Albert R, Assmann SM (2014) Multi-level Modeling of Light-Induced Stomatal Opening Offers New Insights into Its Regulation by Drought. PLoS Comput Biol 10(11): e1003930. doi:10.1371/journal.pcbi.1003930

Sun et al. [15] recently constructed a signal transduction network based on conclusions from more than 85 articles in the literature, describing how more than 70 nodes (proteins, small molecules, ions) interact with each other in the stomatal opening process. The network, reproduced as Fig. 1 [15], includes four source nodes that correspond to the signals red light, blue light, CO_2_, and ABA. The more than 150 edges are directed and signed, with arrowheads indicating activation and terminal black circles indicating inhibition.

Translating this network into a dynamic model, Sun et al. characterized each node with a discrete variable describing its activity and with a discrete (logical) regulatory function describing its regulation. Twenty-one out of the 70 nodes in the model are multi-level, the rest are Boolean (binary). The levels reflect relative and qualitative information: a level of 2 is a higher level than 1, but should not be interpreted as twice as high. A few discrete values are not integers; e.g. stomatal opening is a weighted sum with non-integer weights. The dynamic model has ~10^31^ states. The logical regulatory functions, describing each node’s future state based on the states of the node’s regulators, use a combination of Boolean logic operators (*And, Or, Not*), algebraic operations, and input-output tables. For example, the regulatory function of PRSL1 is:

$$ \mathbf{PRSL}{\mathbf{1}}^{*} = \mathbf{phot}{\mathbf{1}}_{\mathbf{complex}} Or\kern0.5em \mathbf{phot}\mathbf{2}. $$

Here for simplicity the node states are denoted by the node names; the asterisk in “PRSL1*” indicates that this will be the next state of the PRSL1. The “***Or***” Boolean operator expresses that either of the blue light receptors, i.e. the phot1 complex or phot2, can independently activate PRSL1.

The Sun et al. model starts from an initial condition representative of closed stomata. Then a combination of the four input signals is applied. Red light, blue light, and ABA are represented as binary variables, and external CO_2_ is represented with three states: 0 (CO_2_ free air), 1 (ambient CO_2_) and 2 (high CO_2_). The system’s response is simulated through repetitive re-evaluation of each node’s state until a stable value of stomatal opening is observed. The model successfully captures stomatal opening in response to combinations of the signals. It also successfully reproduces stomatal opening under most of the experimentally studied perturbation scenarios (i.e. genetic knockouts or external supply of components). In total, the model is consistent with 63 out of 66 experimental observations collected by Sun et al. [15]. The model predicts the outcome of a large number of scenarios that have not been explored experimentally so far. It also revealed a gap of knowledge regarding the cross-talk of red light and ABA signaling, and filled it with a newly predicted interaction.

Although the Sun et al. model recapitulates existing knowledge and offers new predictions, the model’s full dynamic repertoire could not be characterized due to its large state space. Instead, Sun et al. focused on tracking the output node, stomatal opening, and a few selected internal nodes, in time. In this paper we apply multiple methods to analyze the model and aim to fully map all its potential long-term behaviors, or in other words, attractors.

## Methods

### Attractors of a dynamical system

An attractor is a set of states from which only states in the same set can be reached. Attractors that consist of a single state are called stable steady states or fixed points; attractors that contain multiple states are called complex attractors or oscillations [10]. In biological networks, attractors often have significant biological meaning. In a cell signaling network, attractors correspond to cell types, cell fates or behaviors [21]. For example, one attractor can represent a healthy differentiated cell, while another attractor can represent an abnormally motile cancer cell [22].

### Update scheme of a discrete time model

In the Sun et al. model, as in most discrete dynamic models, time is an implicit variable. As there is very little information about the kinetics of the nodes in the stomatal opening network, the model incorporates an element of stochasticity in timing. The timing does not affect a system’s fixed point attractors, but it can change the complex attractors and the possibility of reaching a given attractor from a given initial state [10]. In the Sun et al. model, a random–order asynchronous update is used. Specifically, at each time step, a random order of nodes (excluding the four input nodes and the output node stomatal opening) is generated, and each node’s state is reevaluated in this order; stomatal opening is always updated last. In the next time step a different order is generated randomly. In this paper, we use a different type of stochastic update, called general asynchronous update, wherein a randomly selected node is updated at each time step. This is required by the network reduction method we use. Although this theoretically could cause a difference in complex attractors, we will show that in this specific model the two update methods yield the same attractors.

### Network reduction

To reduce the Sun et al. model’s state space, we apply a network reduction method developed by Saadatpour et al. [23] that is proven to preserve the attractors of a Boolean model. Two types of nodes can be reduced (eliminated or merged): source nodes with no incoming edges, and simple mediator nodes that have one incoming and/or one outgoing edge. In the reduction, the source node’s state is directly plugged into the regulatory function of all of its direct successor nodes; then the source node is eliminated. For a simple mediator node with one predecessor (regulator) and one successor (target), its regulator is connected to its target and the mediator node is merged into the regulator. If there is one regulator and several targets of the mediator node, but no direct edges between the regulator and any of the targets, the mediator node is merged into the regulator. Conversely, if there are several regulators and one target of the mediator node, but no direct edges among any of the regulators and the target, the mediator node is merged into its target. Although this method is not proven in the multi-level case, we conjecture that attractors are also conserved for a multi-level model, and will show from the results that in the Sun et al. model this reduction method preserved all attractors.

### Elimination of redundant edges

During the process of creating a discrete dynamic model from biological data, when an influence is weaker than other influences, the modeler may choose to omit this influence or, alternatively, include it a redundant way. The latter choice was made by Sun et al. in four cases, leading to four regulatory functions that contain an input that does not affect the outcome of the regulatory function. One of these is

$$ \mathbf{R}\mathbf{O}{\mathbf{S}}^{*} = \mathbf{NADPH}\kern0.5em  And\kern0.5em \mathbf{AtrbohD}/\mathbf{F}\kern0.5em  Or\kern0.5em \mathbf{NADPH}\kern0.5em  And\kern0.5em \mathbf{AtrbohD}/\mathbf{F}\kern0.5em  And\kern0.5em \mathbf{CDPK}\kern0.5em  Or\kern0.5em  Not\kern0.5em \mathbf{Atnoa}\mathbf{1} $$

The italicized words “***And***”, “***Or***” and “***Not***” are Boolean logic operators; the non-italicized words represent node names. In this regulatory function every node is Boolean (binary). The first clause “**NADPH*And*AtrbohD/F**” and the second “**NADPH*And*AtrbohD/F*And*CDPK**” are connected with an “***Or***” rule, with the result that the node “CDPK” does not have any influence on the outcome. Therefore, we can prune the edge from CDPK to ROS without changing the model’s dynamics. We similarly prune three additional redundant edges.

### Converting a multi-level model to Boolean

There are several possibilities to convert a multi-level model to Boolean [24]. The standard method used in the case of logical models of regulatory networks is the Van Ham mapping [25, 26]. It preserves the dynamics of the original model if the variables in the original model can be represented by integers and if the original model only allows state transitions in which one node changes its state by one level [26]. The Sun et al. model does not satisfy these criteria. However there still is a conclusion that we can use: All types of conversions maintain the fixed points and the reachability of states (i.e. if there is a sequence of state transitions from state A to state B before conversion, there must be a sequence of state transitions from the corresponding state A’ to state B’ after the conversion) [26]. So the worst distortion of attractors due to the conversion is the merging of two complex attractors into one. In this light we choose to use an economic mapping of each multi-level node into as many Boolean nodes as necessary for the binary representation of the corresponding integer. We will show that in this specific model, the conversion did not change the attractors.

### Abbreviations

Table 1 summarizes the full names of the network components denoted by abbreviated node names in Fig. 1. The same abbreviations are used in the original Sun et al. model and the reduced model developed in this paper.

**Table 1:** Full names of the network components denoted by abbreviated node names in Fig. 1

Abbreviation	Full name	Abbreviation	Full name
14-3-3 protein_H-ATPase_	14-3-3 protein that binds to the H^+^-ATPase	14-3-3 protein_phot1_	14-3-3 protein that binds to phototropin 1
ABA	abscisic acid	ABI1	2C-type protein phosphatase
acid. of apoplast	the acidification of the apoplast	AnionCh	anion efflux channels at the plasma membrane
AtABCB14	ABC transporter gene AtABCB14	Atnoa1	protein nitric oxide-associated 1
AtrbohD/F	NADPH oxidase D/F	AtSTP1	H-monosaccharide symporter gene AtSTP1
Ca^2+^-ATPase	Ca^2+^-ATPases and Ca^2+^/H+ antiporters responsible for Ca^2+^ efflux from the cytosol	CaIC	inward Ca^2+^ permeable channels
CaR	Ca^2+^ release from intracellular stores	carbon fixation	light-independent reactions of photosynthesis
CDPK	Ca^2+^-dependent protein kinases	CHL1	dual-affinity nitrate transporter gene AtNRT1.1
C_i_	intercellular CO_2_ concentration	FFA	free fatty acids
H^+^-ATPase	the phosphorylated H^+^-ATPase at the plasma membrane prior to the binding of the H^+^-ATPase 14-3-3 protein	H^+^-ATPase_complex_	14-3-3 protein bound H^+^-ATPase
KEV	K^+^ efflux from the vacuole to the cytosol	K_in_	K^+^ inward channels at the plasma membrane
K_out_	K^+^ outward channels at plasma membrane	LPL	lysophospholipids
NADPH	reduced form of nicotinamide adenine dinucleotide phosphate	NIA1	nitrate reductase
NO	nitric oxide	OST1	protein kinase open stomata 1
PA	phosphatidic acid	PEPC	phosphoenolpyruvate carboxylase
phot1	phototropin 1	phot1_complex_	14-3-3 protein bound phototropin 1
phot2	phototropin 2	Photophos-phorylation	light-dependent reactions of photosynthesis
PIP2_C_	phosphatidylinositol 4,5-bisphosphate located in the cytosol	PIP2_PM_	phosphatidylinositol 4,5-bisphosphate located at the plasma membrane
PLA_2_β	phospholipase A2β	PLC	phospholipase C
PLD	phospholipase D	PMV	electric potential difference across the plasma membrane
PP1_cn_	the catalytic subunit of type 1 phosphatase located in the nucleus	PP1_cc_	the catalytic subunit of type 1 phosphatase located in the cytosol
protein kinase	a serine/threonine protein kinase that directly phosphorylates the plasma membrane H-ATPase	PRSL1	type 1 protein phosphatase regulatory subunit 2-like protein1
RIC7	ROP-interactive CRIB motif-containing protein 7	ROP2	small GTPase ROP2
ROS	reactive oxygen species	[Ca^2+^]_c_	cytosolic Ca^2+^ concentration
[Cl^−^]_c/v_	cytosolic/vacuolar Cl^−^ concentration	[K^+^]_c/v_	cytosolic/vacuolar K^+^ concentration
[malate^2−^]_a/c/v_	apoplastic/ cytosolic/vacuolar malate^2−^ concentration	[NO_3_^−^]_a/c/v_	apoplastic/cytosolic/vacuolar nitrate concentration

## Results

### Network reduction

The Sun et al. model has a huge state space of ~10^31^ states, making its analysis difficult. To obtain a smaller state space, we reduce the size of the network by applying a network reduction technique developed by Saadatpour et al. [23] that is proven to preserve the attractors of Boolean models (see Methods). All source nodes other than the four signals (blue light, red light, CO_2_, and abscisic acid) and all simple mediator nodes are identified and reduced. This process is done iteratively until it cannot be done any more. A total of 7 source nodes (14-3-3 protein_phot1_, PIP2_C_, AtNOA1, Nitrate, PP1_cn_, mitochondria, and CHL1), and 19 simple mediator nodes (phot1, phot2, NIA1, H^+^-ATPase, LPL, ATP, acid. of apoplast, [NO_3_^−^]_v_, [Cl^−^]_v_, NADPH, [malate^2−^]_v_, PA, ABA receptors, OST1, PRSL1, PIP2_PM_, AtrbohD/F, Nitrite, and phot1_complex_) are eliminated. Several of the simple mediator nodes form linear paths (e.g. phot1, OST1) thus their iterative reduction shortens the linear paths in the network. In addition, 16 of the 19 reduced mediators have a regulatory function of the form “B* = A”. It is intuitive that reduction of this node type preserves the attractors.

We do not eliminate the four signal nodes because we want to simultaneously explore all the combinations of input signals. We also choose to not reduce the five nodes (K_in_, K_out_, K_c_, Ca^2+^-ATPase, mesophyll cell photosynthesis) whose merging with their sole regulator would result in a self-loop (self-regulation), because such self-loops may be difficult to interpret. Two additional nodes with significant biological meaning to the network (sucrose, stomatal opening), are not reduced either.

Another form of network reduction is the elimination of redundant edges (see Methods). After removal of redundant edges, the node CDPK becomes a sink node, thus it can also be eliminated. The reduction of the above-described nodes and redundant edges simplifies the network from 70 nodes to 42 nodes, with an estimated state space of ~10^22^ states.

### Simplification of regulatory functions

In order to further reduce the state space from ~10^22^ to a manageable size, we grouped state values so that nodes are represented with fewer states. This grouping was guided by the 66 experimental observations summarized in Sun et al.; we aimed to maintain the reduced model’s results consistent with these experimental observations.

For example, in the Sun et al. model [15] the regulatory function of Stomatal Opening is a weighted sum of different ions and sucrose:

$$ \begin{array}{l}\mathbf{Stomatal}\kern0.5em \mathbf{openin}{\mathbf{g}}^{\ast }={\left[\mathbf{C}{\mathbf{l}}^{-}\right]}_{\mathbf{v}}\mathbf{contribution}+{\left[\mathbf{N}{{\mathbf{O}}_{\mathbf{3}}}^{-}\right]}_{\mathbf{v}}\mathbf{contribution}+{\left[{\mathbf{K}}^{+}\right]}_{\mathbf{v}}+\\ {}{\left[\mathbf{malat}{\mathbf{e}}^{\mathbf{2}-}\right]}_{\mathbf{v}}\mathbf{contribution}+\mathbf{sucrose}-\mathbf{R}\mathbf{I}\mathbf{C}\mathbf{7}/\mathbf{6}\end{array} $$

The weights of the anion contributions to the osmotic potential were chosen based on the literature. Also, the anion contributions must not exceed a proportion of [K^+^]_v_ due to charge balance. The anion contributions are [malate^2−^]_v_ contribution ≤ 0.425 × [K^+^]_v_; [NO_3_^−^]_v_ contribution ≤0.10 × [K^+^]_v_; [Cl^−^]_v_ contribution ≤ 0.05 × [K^+^]_v_. The primary contributions come from [K^+^]_v_ and sucrose. We grouped the stomatal opening values into 6 groups with different [K^+^]_v_ and sucrose values (see Table 2 and Additional file 1).

**Table 2:** Grouping of the stomatal opening values by the level of [K^+^]_v_ and sucrose

[K^+^]_v_	Sucrose	Stomatal opening value in the Sun et al. model	Simplified stomatal opening value
0	0	0	0
0	1 or 2	1 or 2	1
1	0	1.58	1
1.8	1	3.84	2
1.5	2	4.36	2
2	0 or 1	3.15 or 4.15	3
4.5	0 or 2	5.18 or 8.92	3
6	0	9.28 or 9.45	5
6	2	11.28 or 11.45	5
9	0 or 2	14.01 or 16.01	6

The first two columns indicate the [K^+^]_v_ and sucrose levels. The third column is the possible values of stomatal opening in the Sun et al. model for the given [K^+^]_v_ and sucrose levels. Note that here we only show [K^+^]_v_, sucrose and stomatal opening value combinations observed in the simulations of the 66 experimentally studied scenarios reported by Sun et al. [15]. More stomatal opening values are possible when considering node perturbations. The 4th column shows the simplified stomatal opening level after grouping. The update function for the simplified stomatal opening level covers all possible values of [K^+^]_v_ and sucrose (see Additional file 1).

Similarly to the original model, the simplified states represent qualitative, relative categories. For example, a stomatal opening level of 2 is not twice as high as level 1. We choose the simplified stomatal opening values so that there is no state “4”, to better reflect an experimentally observed synergistic effect between blue and red light [18, 19, 27]. Simulation results with the simplified regulatory function are that under monochromatic red light stomatal opening =1; under monochromatic blue light stomatal opening =3; under dual beam the stomatal opening =5, which is larger than the sum “1 + 3”. This qualitatively reproduces the experimental observation that under dual beam illumination stomata open to a size much larger than the sum of opening under monochromatic blue or red light.

We find by simulation of the reduced model, using the same initial condition as the Sun et al. model, that the simplification of the stomatal opening regulatory function results in only 3 additional cases of inconsistency with experimental observations out of a total of 66 experimentally studied scenarios. Additional file 2 lists all experimental observations and compares them to the relevant simulation results. Ignoring the contribution of malate^2−^, NO_3_^−^, and RIC7 to stomatal opening each causes one additional discrepancy; ignoring Cl^−^ does not cause any additional discrepancy. Ignoring these nodes trades a decrease in accuracy for a significant increase in simplicity.

**Fig. 2:**
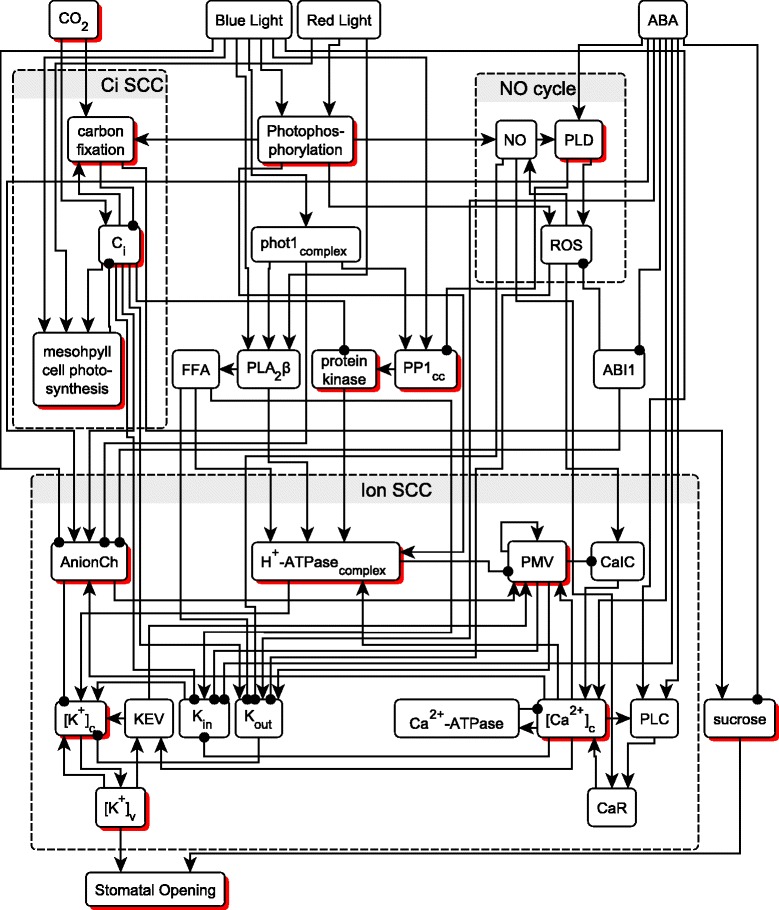
The stomatal opening network after model reduction, with 32 nodes and 81 edges. Nodes with shadows have multiple states; other nodes are binary. The three strongly-connected components (SCCs) of the network are indicated by rectangles with dashed contours

The simplification of the stomatal opening regulatory function eliminates the effect of vacuolar anions and of RIC7 on stomatal opening. As a result we can further simplify the Sun et al. model by eliminating 10 nodes in total, [malate^2−^]_a_, [malate^2−^]_c_, starch, [Cl^−^]_c_, [NO_3_^−^]_c_, [NO_3_^−^]_a_, ROP2, RIC7, ABC, and PEPC. The only edge from these nodes to other nodes is [malate^2−^]_a_ → AnionCh. In section 3 of Additional file 3 we show that eliminating this edge does not change the system’s long-term behavior, i.e. attractors. Also, the regulatory function describing the cytosolic K^+^ concentration, [K^+^]_c_, can be simplified without loss, as described in section 3 of Additional file 3. After this simplification we have a network of 32 nodes, 81 edges, indicated on Fig. 2. We will refer to this model as the “reduced model”. A list of nodes and their regulatory functions is provided in Additional file 1.

Identifying strongly connected components (SCCs) is important for attractor analysis, as complex dynamic behavior such as oscillations or multi-stability requires feedback loops [7]. There are three SCCs in the network of the reduced model, as marked in Fig. 2. The NO cycle contains three nodes and three positive edges. The C_i_ SCC contains three nodes, which form two negative feedback loops. The Ion SCC is the most complex, containing 13 nodes and 26 edges, 7 of which are negative.

Next we perform attractor analysis using two methods: 1. by converting the reduced model to Boolean and applying two analysis tools; 2. by analyzing the regulatory functions theoretically. The former method finds all stable steady states and candidate oscillations; the latter confirms the results of the first method and gives insight about perturbation scenarios.

### Conversion of nodes from multi-level to Boolean states and attractor analysis

We perform the conversion to Boolean to enable attractor analysis by existing software tools. Zañudo et al. [28] proposed an algorithm to find the attractors of a Boolean network based on the concept of “stable motif”, a strongly-connected group of nodes that can stabilize regardless of their inputs. The algorithm finds all stable motifs, which determine the part of the network that stabilizes in an attractor. After a stable motif is found, one can plug in its stabilized state into the network, and obtain a smaller remaining network. After repeating this, eventually the remaining part is either nothing (indicating a fixed point/stable steady state) or a candidate oscillating sub-network. Compared with other software tools [29, 30], the major advantage of this algorithm is that it finds all the attractors of Boolean networks with hundreds of nodes [28]. Application of this powerful method requires a Boolean model, so we convert the multi-level model into Boolean first (see Methods). An example of conversion is given in Table 3.

**Table 3:** Example of Boolean conversion

Level of the original node	State of Boolean node_2	State of Boolean node_1
0	0	0
1	0	1
2	1	0
3	1	1

The multi-level node shown in the 1st column is mapped into two Boolean nodes, shown in the 2nd and 3rd columns, using the binary representation of the corresponding integer.

More detailed examples of the conversion of the states and regulatory function of specific nodes are given in the Additional file 4. We will refer to the reduced model after conversion to Boolean variables as the “Boolean-converted reduced model”. The regulatory functions of the Boolean-converted reduced model are available in Additional file 5. When simulating the Boolean-converted reduced model, all the Boolean nodes that represent the same entity (the same multi-level node) are updated simultaneously. In this way the state transitions of the reduced model will be kept the same in the Boolean-converted reduced model, and therefore the Boolean conversion will not cause additional discrepancies from experimental observations.

We apply the stable motif algorithm’s implementation, downloaded from http://github.com/jgtz/StableMotifs/ [28], to the Boolean-converted reduced model. The algorithm uses the Boolean regulatory functions of the converted model (given in Additional files 5 and 6) as input. We consider every combination of sustained states of the five signal nodes (blue light, red light, ABA, CO_2_, CO_2__high). We find two possible stable motifs, corresponding to the self-regulatory node PMV_pos (one of the two Boolean nodes associated with the multi-level node PMV, see Additional files 4 and 5), in conditions where the H^+^-ATPase_complex_ is inactive. These two stable motifs indicate the bistability of PMV. Under its influence, another node, K_out_, will also be bistable. The algorithm also indicates that for any signal combination, every node, except [Ca^2+^]_c_ and Ca^2+^-ATPase, will stabilize in a fixed state. [Ca^2+^]_c_ has three states, and in the Boolean-converted model it is represented by two nodes, Cac and Cac_high. Cac_high, which represents the higher level of [Ca^2+^]_c_, stabilizes at zero in all situations. Cac and Ca^2+^-ATPase may oscillate in conditions where blue light is present and ABA is absent (a total of six cases, two of which allow PMV bistability). Table 4 summarizes key features of the attractors found by the stable motif algorithm for all 24 input combinations. Attractors where Ca^2+^ oscillation is not possible are fixed points (stable steady states).

**Table 4:** Summary of the attractors found using the stable motif algorithm

BL	RL	CO_2_	CO_2__high	ABA	SO (Bool)	SO	Ca^2+^ Oscillation Possible?	PMV_pos bistability
0	0	Any	Any	Any	000	0	No	Yes
0	1	0	0	1	000	0	No	No
0	1	1	Any	1	000	0	No	Yes
1	Any	1	0	1	000	0	No	No
1	Any	1	1	1	000	0	No	Yes
0	1	1	Any	0	010	1	No	Yes
1	Any	1	1	0	010	1	Yes	Yes
0	1	0	0	0	101	3	No	No
1	0	1	0	0	101	3	Yes	No
1	Any	0	0	1	101	3	No	No
1	0	0	0	0	110	5	Yes	No
1	1	1	0	0	110	5	Yes	No
1	1	0	0	0	111	6	Yes	No

The first five columns indicate the input signal combination. The setting CO_2__high = 1 and CO_2_ = 0 is not included because it is not biologically meaningful. The “SO (Bool)” column indicates the state of the Boolean node combination representing stomatal opening. The “SO” column is the state of stomatal opening when converted back to an integer. Note that the stomatal opening level of four is not defined, and no attractors have a stomatal opening level of two. The next column indicates whether Ca^2+^ oscillation can possibly happen under the given signal combination. The last column indicates whether bistability of PMV_pos can be observed under this setting. In those cases, two stable steady states with (PMV_pos = 0, K_out_ = 0) and (PMV_pos = 1, K_out_ = 1) can be observed. The rest of the nodes are unaffected by this two-node bistability

We verified the obtained attractors with GINsim [12], a software suite capable of model construction, simulation, and analysis. GINsim can compute all stable steady states (called stable states in GINsim), or determine complex attractors by mapping the state transitions. The stable steady states found by GINsim are identical to those found by the stable motif algorithm. To verify and further explore the complex attractors, we use the simulation function of GINsim, starting from a state in the complex attractor. The result that the system oscillates between four states, where only the state of Cac and Ca^2+^-ATPase changes, agrees with the findings of the stable motif algorithm. We summarize the GINsim computation/simulation results in Additional file 7. Additional file 8 indicates the Boolean-converted reduced model in SBML-qual format [29], a general format for biological model to be analyzed using various tools including GINsim.

We can also connect the stable motif analysis results to network reduction. We have previously decided to not reduce the four nodes that correspond to input signals. If we do consider a specific input combination when using network reduction, e.g. blue light and red light with normal CO_2_ without ABA, we can reduce much more of the network: two of the three SCCs, namely the NO cycle and the C_i_ SCC, will stabilize and can be eliminated. Only the Ion SCC and its sole output stomatal opening remain, indicating that this SCC is not driven solely by the external signals and has the capacity for oscillations or multi-stability. This is consistent with the results found by stable motif analysis, according to which the NO cycle and the C_i_ SCC attain a steady state and the Ion SCC admits a [Ca^2+^]_c_ - Ca^2+^-ATPase oscillation and PMV bistability. This consistency supports the appropriateness of the network reduction method and of the Boolean conversion.

### Theoretical analysis of the reduced model

To gain additional insight into the attractors of the reduced model and their potential changes due to node perturbations, we analyze the reduced model theoretically. Specifically, we aim to answer the question: Can there be other types of oscillation, or can there be additional multi-stability, if a node is knocked out (fixed in the OFF state) or is constitutive active (fixed in the highest state)?

We first test whether the network and regulatory rules allow multi-stability or oscillations. This analysis is based on R. Thomas’s conjectures [7]: The presence of a positive (negative) feedback loop - a cycle with an even (odd) number of inhibitory edges - in the network is a necessary but not sufficient condition for the occurrence of multiple steady states (oscillations). The conjectures have been proven in the case of discrete dynamic systems [31–34]. Since only feedback loops are candidates for potential multi-stability or oscillations, we analyze the regulatory functions of each strongly connected component of the network. For each feedback loop, we identify a sufficient condition for the nodes to stabilize in a specific state. The violation of this condition becomes a further necessary condition of multi-stability or oscillation. Here we describe the main steps and results of the analysis; the detailed analysis is in Additional file 3.

The NO cycle is composed of the nodes PLD, ROS, NO, and the three positive edges between them. It does not have any negative edges, so it cannot oscillate. A fixed ABA value is sufficient to stabilize each node of the cycle in a specific state, thus the cycle does not admit multi-stability under any perturbation.

The C_i_ SCC has three nodes, C_i_, mesophyll cell photosynthesis (MCPS), carbon fixation, and four edges that form two negative feedback loops, one between carbon fixation and C_i_, and the other between C_i_ and MCPS. Despite the existence of negative feedback, this cycle will stabilize if given a fixed CO_2_ value. From this we know that this cycle cannot oscillate or admit multi-stability under any perturbation.

**Fig. 3:**
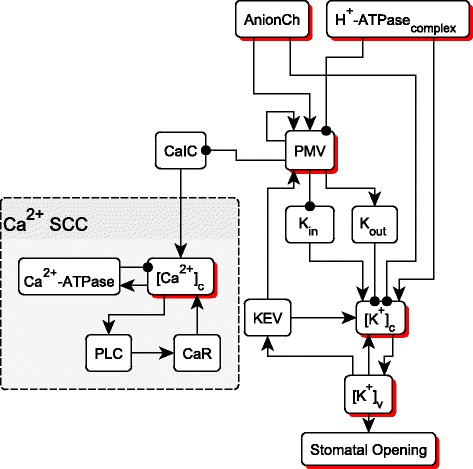
The Ion SCC after reducing all edges that depend on calcium. All regulators of this sub-network have been omitted. On the left, [Ca^2+^]_c_ related nodes form a sink sub-network

The Ion SCC has 13 nodes. To reduce its complexity we show that the key node [Ca^2+^]_c_, which has states 0,1, and 2, cannot enter state 2 in the long term under any perturbation. Since most nodes respond to [Ca^2+^]_c_ only if [Ca^2+^]_c_ =2, we can eliminate all edges that depend only on “[Ca^2+^]_c_ =2”, and obtain a simplified Ion SCC, as shown in Fig. 3. The Ca^2+^ SCC ([Ca^2+^]_c_, Ca^2+^ ATPase, PLC, CaR) now becomes a sink SCC. The only negative edge in this sub-network is from Ca^2+^-ATPase to [Ca^2+^]_c_. These two nodes are known to oscillate. The positive feedback loop formed by [Ca^2+^]_c_, PLC, and CaR will stabilize if given fixed inputs. So there cannot be multi-stability. For the nodes outside of the Ca^2+^ feedback loops, we show that the edges from KEV and [K^+^]_v_ are redundant in the long term, so there are no feedback loops except the PMV self-loop. PMV is not capable of having oscillations, but can have bistability (as also indicated by the stable motif analysis). The bistability can affect at most one other node, K_out_, under any perturbation. This means that the bistability has very limited effect on the attractor of the reduced model.

Now we can summarize our conclusions and return to the question we sought to answer: there is no oscillation except in the calcium nodes; there is no multi-stability except in the nodes PMV and K_out_. These statements are true under any perturbation. Moreover, for the calcium oscillation, [Ca^2+^]_c_ cannot enter the state 2, so the sub-network between [Ca^2+^]_c_ and Ca^2+^-ATPase is a negative feedback loop between two Boolean nodes, with the regulatory functions Ca^2+^ ATPase^*^ = [Ca^2+^]_c_; [Ca^2+^]_c_* = not Ca^2+^ ATPase. It results in the simplest type of oscillation, as also found by GINsim simulation. For the PMV bistability, even if the bistability exists, most nodes, especially the output node stomatal opening, still have a unique value. Thus the theoretical analysis, in agreement with the computational analysis, leads to very strong conclusions about the reduced model’s dynamic repertoire.

We can also show that the reduction or Boolean conversion did not change the attractors of the Sun et al. model. Although the reduction we used is only proven in the Boolean case, Naldi et al. showed that for multi-valued models, removal of non-autoregulated nodes, like in our reduction, preserves crucial dynamical properties [35], including fixed point attractors and the two-node simple oscillation we found. So our reduction is valid in this specific model. To confirm that the Boolean conversion preserved attractors, we note that in the Boolean-converted reduced model we found fixed point attractors and a complex attractor in which only two nodes oscillate. Because the only potential change to attractors as a consequence of the conversion is merging of complex attractors [26], it is straightforward that the attractors have been conserved during the conversion, as the two-node oscillation found is the simplest type of complex attractor and cannot be a result of attractor merging. In addition, using general asynchronous update instead of random order asynchronous update does not cause any changes to the attractor, because the update schemes do not affect fixed points or the two-node simple oscillation we found.

### Stability of guard cell signal transduction

Our previous results indicate the stability of the system in the sense that all the initial conditions lead to the same attractor except for up to four nodes. We also examine another facet of the system’s stability: the robustness of the stomatal opening in response to node perturbations that render them non-functional. We perform a systematic analysis of single-node knockouts of every non-signal node in the reduced model, under all combinations of light, CO_2_ and ABA conditions. For each signal combination, we set the perturbed node’s initial state and regulatory function to 0, initialize the rest of the nodes in the condition representative of closed stomata, and then simulate the reduced model until it reaches its attractor. In the absence of ABA under each light and CO_2_ condition, 60–90 % perturbation scenarios produce the same stomatal opening value as the unperturbed system (Table 5). These results are similar to those reported by Sun et al. for the original model [15] (see Additional file 9). In the presence of ABA 50–90 % perturbation scenarios produce the same stomatal opening value as the unperturbed system, and 4–16 % knockouts lead to a higher stomatal opening value. Perturbations in the ABA = 1 case were not studied by Sun et al., but our simulations of the original model give the same qualitative results as the reduced model. These results indicate the closeness of the perturbed attractor (at least in terms of the stomatal opening value) to the unperturbed attractor in more than 50 % of single node perturbations. They also suggest the resilience of the stomatal opening process against internal failures and perturbations.

**Table 5:** Summary of systematic perturbation results

Light, CO_2_ and ABA condition			Unperturbed SO level	Simplified SO level						Percentage of cases with unchanged SO value
				0	1	2	3	5	6	
				Percentage of single knockouts that lead to each SO level						
Dual Beam	Mod. CO_2_	ABA OFF	5		4 %		31 %	65 %		65 %
	Low CO_2_		6	31 %			4 %		65 %	65 %
	High CO_2_		1	4 %	96 %					96 %
Blue Light	Mod. CO_2_		3		35 %		65 %			65 %
	Low CO_2_		5	31 %			4 %	65 %		65 %
	High CO_2_		1	4 %	96 %					96 %
Red Light	Mod. CO_2_		1	4 %	96 %					96 %
	Low CO_2_		3	35 %			65 %			65 %
	High CO_2_		1	4 %	96 %					96 %
Dual Beam	Mod. CO_2_	ABA ON	0	85 %		4 %	8 %	4 %		85 %
	Low CO_2_		3	46 %			50 %		4 %	50 %
Blue Light	Mod. CO_2_		0	85 %	4 %	8 %	4 %			85 %
	Low CO_2_		3	46 %			50 %	4 %		50 %
Red Light	Low CO_2_		0	96 %			4 %			96 %

The first set of columns, with the header ‘Light, CO_2_ and ABA condition’, indicate the input signal combinations. The abbreviation “Mod.” means moderate CO_2_ concentration. Note that we do not list the four input combinations (high CO_2_ with ABA and with any type of light, or moderate CO_2_ with ABA and red light) wherein all simulated stomatal opening values are zero. The 2nd column is the simulated stomatal opening (SO) level in the unperturbed system. The 3rd column set shows the percentage of single-node knockouts that yield the corresponding SO level. There is no stomatal opening level 4 in the reduced model. No entry means zero percentage. The last column is the percentage of settings where the stomatal opening remains at the same level as the unperturbed case. A complete table of perturbation results is provided in Additional file 9

### Extending the conclusions to the original model

We found that in the reduced model there is no oscillation except in the calcium nodes; there is no multi-stability except in the nodes PMV and K_out_. Because the reduction we used has been shown to conserve attractors [23, 35], we know that our attractor conclusions can be immediately extended to all nodes in the original model except the reduced nodes and stomatal opening. Next we extend the attractor analysis to include the reduced nodes as well.

First we consider the nodes reduced during the first step of network reduction, i.e. non-signal source nodes and simple mediator nodes. These nodes are trivially incapable of having multi-stability and oscillations themselves, so we need only to consider their perturbations. Perturbation of a simple mediator node can always be replaced by a corresponding (set of) perturbation(s) in the mediator node’s direct successor(s), so these perturbations have already been considered. Perturbing a non-signal source node may theoretically cause a difference, however the nodes in this category in the Sun et al. model represent molecules that are abundant in the cell or cell environment, thus their perturbation is not biologically relevant or practical.

Next we consider the anion nodes reduced due to the simplified stomatal opening rule. Recall that these nodes do not affect other nodes except stomatal opening in the long term. There cannot be multi-stability in anion nodes unless the assumptions of sufficient initial [NO_3_^−^]_a_ and starch concentration, and sufficient initial mitochondrial TCA cycle activity are violated (details are provided in Additional file 3, section 5 and 6). Since there is no support for interventions that would lead to the violation of these assumptions, it is reasonable to conclude that no multi-stability can be found in the reduced nodes under biologically relevant situations. We also found that there can be an additional oscillation in the RIC7 path (involving the nodes ROP2, RIC7 and SO) when a special set of perturbations is applied. Under that case, the nodes RIC7 and SO will oscillate. Since the effect of this behavior is small (within 5 % of the unperturbed SO value in the Sun et al. model [15]), it has little biological significance. There are no more possible oscillations as there are no more negative feedback loops. To conclude, the original Sun et al. model has oscillations only in cytosolic Ca^2+^ ([Ca^2+^]_c_) and Ca^2+^ ATPase, and has multi-stability only in PMV and K_out_, under situations that are biologically meaningful.

## Discussion

The conclusions we obtained can tell us how to control this network model. Generally in engineering applications, control means to drive a system into an arbitrary state [36, 37]. However in biological systems, it is more meaningful to drive the system into one of its natural attractors rather than into an arbitrary state, as the attractors correspond to stable phenotypes [38]. To control the attractor of a Boolean system, one needs to control only its input nodes and a subset of nodes in each stable motif [39]. Our integrated analysis, involving Boolean conversion, indicates that to control the attractor that the stomatal opening network evolves into, one only needs to control the input signals and PMV, even in case of perturbations. In particular, to control the stomatal opening value, one only needs to control the input signals, under any perturbation.

The reduced model provides new biological insights. Normally, when ABA is present, stomata will close. However in some knockout mutants stomata can open to a certain extent in the presence of ABA, although the opening level is not as much as in the case without ABA [15]. Such partial reversals of the effect of ABA are important for understanding the mechanism of stomatal opening. For example, Sun et al. reported that OST1 knockout (OST1 is kept 0) and inhibition of the NADPH oxidase (AtrbohD/F is kept 0) yielded partially restored SO level in simulations, in agreement with experimental observations (see Additional file 2 for the comparison of the equivalent simulations in the reduced model with experiments). Simplification of the Sun et al. model allows easier simulation of more perturbation scenarios, e.g. the systematic identification of possible partial reversals. Table 6 indicates all the partial reversals due to single node knockouts in the reduced model.

**Table 6:** Nodes whose knockouts diminish ABA’s inhibition of stomatal opening

Light, CO_2_ and ABA condition		Unperturbed SO level	Nodes whose knockout results in a partially restored SO, and the corresponding SO value				
			CO_2_	NO	PLD	ROS	AnionCh
Dual Beam	Moderate CO_2_, ABA is present	0	3	3	5	3	2
Blue Light		0	3	2	3	2	1
Red Light		0			3		1

The first set of columns, with the header ‘Light, CO_2_ and ABA condition’, indicate the input signal combinations. The 2nd column is the stomatal opening without perturbations. The 3rd column set indicates the nodes whose knockout would yield a stomatal opening level that is higher than the unperturbed value of 0. CO_2_ knockout means CO_2_ being set to zero (CO_2_ free air). No entry means the setting does not cause partial reversal

Our results reproduce the observation that knockout of nodes in the ABA pathway (PLD, NO, ROS) can cause partial reversals of ABA’s effect. We find that AnionCh knockout can partially restore stomatal opening inhibited by ABA, a result not reported by Sun et al., but which is supported by experimental evidence [40]. In addition, Table 6 offers a new biological prediction: low CO_2_ concentration can partially restore stomatal opening when ABA is present. This is consistent with the knowledge that CO_2_-free air promotes stomatal opening in the absence of ABA [41]. This CO_2_ effect suggests a mechanism of cross-talk between CO_2_ and ABA. Importantly, apart from the five nodes listed in Table 6, no other node’s knockout can reverse ABA’s inhibition of stomatal opening. The perturbation results of Table 5 offer many more new predictions.

Our combination of techniques offers a powerful framework for determining the dynamic repertoire of a multi-level dynamic model. Multi-level models are more accurate than Boolean models in describing the quantitative characteristics of dynamic systems, but there are few general methods to analyze multi-level models [10, 12]. By combining different existing methods, we were able to overcome the limitations of each method. Our successful combination of existing methods offers a promising way to analyze multi-level models, and might point towards a general strategy to analyze the attractors of multi-level models, biological or non-biological.

A notable future direction for this work is to develop an alternative way to determine the attractors of multi-level models by extending the concept of stable motifs. Compared with conversion to a Boolean model, then applying Boolean stable motif algorithm, extending the stable motif algorithm to multi-level models can avoid potential attractor change issues. Development of such a technique will allow easy and powerful attractor analysis for multi-level models.

## Conclusions

We obtained a very strong conclusion about the attractors of the Sun et al. stomatal opening model: under any combination of sustained signals, all nodes in the model converge into steady states, with the potential exception of the cytosolic Ca^2+^ ([Ca^2+^]_c_) and Ca^2+^ ATPase. Variations in the initial condition of non-source nodes or in process timing (node update sequence) can drive at most two nodes, PMV and K_out_, into a different attractor. This high degree of attractor similarity is somewhat unexpected, as the network has a large strongly connected component and several feedback loops. Thus, despite the decidedly non-linear structure of the network, most parts of the system behave in the consistent manner of a linear pathway. This is a distinct feature of the stomatal opening model: many dynamic models of biological systems have multiple, diverse attractors [22, 42]. The models of these systems will evolve into drastically different attractors when starting from different initial conditions, sometimes even when starting from the same initial condition, demonstrating different biological trajectories. In the stomatal opening model, however, the uniqueness of the steady state stomatal opening level suggests that the final extent of the stomatal opening response is robust and resilient against changes in initial conditions or in timing. Note that although a change in the initial condition will not change the steady-state opening level, it may change the steady state of PMV and K_out_, and may change how fast the system converges to an attractor.

We also showed that the reduced stomatal opening model does not admit additional, emergent oscillations or multi-stability under any biologically relevant node perturbation (knockout or constitutive activity). We further demonstrate the robustness of the system by examining the stomatal opening level under single node knockouts: in most cases the signals are still likely to propagate and lead to a similar degree of stomatal opening as in the absence of perturbation. This robustness is unlike a single linear pathway, which would be very sensitive to node disruption. We suggest that the role of the strongly connected components in the network could be to provide multiple paths for the signal to propagate, but at the same time not allowing extensive multistability or oscillations. Our innovative combination of existing methods offers a promising way to analyze multi-level models.

## Additional files

Additional file 1: Regulatory functions of the reduced stomatal opening model. (DOCX 38 kb) Additional file 2: Compilation of comparisons between published experimental observations and the reduced model’s results for simulations of the identical conditions. (DOCX 163 kb) Additional file 3: Analysis of stomatal opening model. Detailed derivation of attractor analysis and other statements made in the main article. (DOCX 98 kb) Additional file 4: Examples of converting a multi-level update function to Boolean. (DOCX 38 kb) Additional file 5: Regulatory functions of the Boolean-converted reduced stomatal opening model. (DOCX 25 kb) Additional file 6: Text file of the Boolean-converted reduced stomatal opening model to be used in the stable motif algorithm. The stable motif algorithm and instructions about how to use it can be found from this link: https://github.com/jgtz/StableMotifs. (TXT 4 kb) Additional file 7: GINsim attractor analysis of the Boolean-converted reduced stomatal opening model. (DOCX 42 kb) Additional file 8: Boolean-converted reduced stomatal opening model in SBML format. This file format can be used in various tools, including GINsim. (SBML 199 kb) Additional file 9: Stomatal opening levels for simulated single node knockouts in the simplified model under all input signal combinations. (XLSX 15 kb)
