# Supplementary material for: Analysis of a dynamic model of guard cell signaling reveals the stability of signal propagation
Source: BMC Syst Biol. 2016 Aug 19;10:78. doi: 10.1186/s12918-016-0327-7 (PMC4992220; doi:10.1186/s12918-016-0327-7)
Supplement: Additional file 1: — Regulatory functions of the reduced stomatal opening model. (DOCX 38 kb) [file 12918_2016_327_MOESM1_ESM.docx]

Regulatory Functions of the Reduced Stomatal Opening Model

In this file we provide the regulatory functions for each of the 32 nodes in the reduced stomatal opening model. The following table shows the possible states of these nodes; the node names are the same as in Figure 1 in the main text unless specified.

| Possible node levels | List of nodes |
| --- | --- |
| {0, 1} | Blue light, phot1_complex_, PLC, PLA_2_β, CaIC, CaR, NO, Ca^2+^ATPase^[[1]](#footnote-1)^, FFA, K_in_, K_out_, KEV, Red light, ABA, ABI1, ROS |
| {0, 1, 2} | [Ca^2+^]_c_, CO_2_, photophosphorylation, carbon fixation, PLD, sucrose, MCPS (mesophyll cell photosynthesis) |
| {0, 1, 1.6} | AnionCh |
| {0, 0.5, 1, 2} | C_i_ |
| {–2, –1, 0, 1, 2} | PMV |
| {0, 1, 1.5, 2, 3, 3.5, 4} | PP1_cc_ |
| {0, 0.5, 0.9, 1, 1.5, 2, 3} | protein kinase |
| {0, 0.5, 1, 1.5, 2, 3, 4, 6, 9} | H^+^ ATPase_complex_, [K^+^]_c_, [K^+^]_v_ |
| {0,1,2,3,5,6} | stomatal opening |

The regulatory function’s left hand side refers to the node whose state is evaluated, and the right hand side refers to this node’s regulators. The variables of the regulatory function are node states, which for simplicity are denoted by the node name. The regulatory function specifies the next state of the target node (indicated by the use of an asterisk on the name of the target node) as a function of the current states of its regulators. Four of the nodes are input signals that are assumed to have a sustained expression. Thus their next state equals their current state, which can be expressed by making them self-regulated. For example, the regulatory function for Blue Light is “**Blue Light* = Blue Light**”.

The regulatory functions of most other nodes involve the Boolean logic operators “***And***, ***Or***, ***Not***”; True is interpreted as 1 and False is interpreted as 0. The regulatory function of multi-level nodes also involves algebraic operations like addition “**+**” or multiplication “×”. In these functions the state of Boolean nodes is interpreted as the integers 1 or 0. For example, the Boolean nodes A=True=1 B=False=0, and C=True=1 will yield the algebraic relationships A+B=1 and A+C=2. If a multi-level node, say D, is used in a Boolean logic function, we use clauses like “(D>0)” or “(D=2)” to convert its state to Boolean values. As in the Sun et al model, the regulatory functions of several nodes are indicated as truth tables that summarize the next state of the target node for every combination of the states of its regulators.

Compared with the original model, 15 nodes in the reduced model kept the same regulatory functions, namely CaIC, CaR, FFA, [Ca^2+^]_c_, Ca^2+^ATPase, KEV, PLD, PMV, photophosphorylation, carbon fixation, sucrose, Kin, Kout, [K^+^]_v,_ MCPS, C_i_.

**Blue Light* =Blue Light**

**Red Light* =Red Light**

**ABA* =ABA**

**CO_2_* =CO_2_**

**phot1_complex_* = Blue Light**

**PLC* = Blue Light *Or* ABA *And* [Ca^2+^]_c_**

**PLA_2_β* = (phot1_complex_ *Or* Blue Light *Or* Red Light)**

**CaIC* = ROS *And* (PMV<0)**

**CaR* = NO *Or* PLC**

**NO* = (photophosphorylation>0) *And* ROS**

**[Ca^2+^]_c_* = ((CaIC or CaR) *And Not* Ca^2+^ ATPase) + ABA**

**Ca^2+^ ATPase* = ([Ca^2+^]_c_ >0)**

PP1_cc_ truth table

| **Blue Light** | **phot1_complex_** | **PLD** | **PP1_cc_^*^** |
| --- | --- | --- | --- |
| 0 | 0 | 0 | 2 |
|  |  | 1 | 1.5 |
|  |  | 2 | 1 |
|  | 1 | 0 | 4 |
|  |  | 1 | 3.5 |
|  |  | 2 | 3 |
| 1 | any | 0 | 4 |
|  |  | 1 | 3.5 |
|  |  | 2 | 3 |

Protein kinase truth table:

| **C_i_** | **PP1_cc_** | **protein kinase^*^** |
| --- | --- | --- |
| any | 0 | 0 |
| 0 | 1, 1.5 | 0.5 |
|  | 2 | 1 |
|  | 3, 3.5 | 1.5 |
|  | 4 | 3 |
| 0.5 | 1, 1.5 | 0 |
|  | 2 | 0.5 |
|  | 3 | 0.5 |
|  | 3.5 | 1.5 |
|  | 4 | 2 |
| 1 | 1, 1.5, 2 | 0 |
|  | 3 | 0.5 |
|  | 3.5 | 0.9 |
|  | 4 | 1 |
| 2 | any | 0 |

**H^+^ ATPase_complex_*= ((FFA *Or* PLA_2_β) *And Not* ([Ca^2+^]_c_ = 2)) × PK × (1 + photophosphorylation)**

**FFA* = PLA_2_β**

**PMV* = PMV- (H^+^ ATPase_complex_>0) + (AnionCh *And* (PMV<0)) + (([Ca^2+^]_c_ = 2) *Or* KEV)**

**Kin* = (FFA *Or Not* [Ca^2+^]_c_=2 *Or* ABA) *And* (*Not* (Ci==2)) *And* (PMV<0)**

**Kout* = (ABA *Or* (Ci=2) *Or* (*Not* ROS) *Or Not* NO *Or Not* FFA) *And* (PMV>0)**

**[K^+^]_c_* = [(Kin *Or* KEV *And* [K^+^]_v_) *And Not* Kout] ×(H^+^-ATPase_complex_ ≥ AnionCh) ×H^+^ ATPase_complex_**

**KEV* = ([Ca^2+^]_c_ =2) *And* [K^+^]_v_)**

**[K^+^]_v_* = [K^+^]_c_**

**sucrose* = carbon_fixation *And* *Not* ABA**

Ci truth table:

consumption = max{carbon_fixation, MCPS}.

| **CO_2_** | **consumption** | **C_i_^*^** |
| --- | --- | --- |
| 0 (CO_2_-free air) | Any | 0 |
| 1 (moderate atmospheric CO_2_) | 0 or 1 | 1 |
|  | 2 | 0.5 |
| 2 (high atmospheric CO_2_) | Any | 2 |

**photophosphorylation* = Blue Light + Red Light**

**Carbon_fixation* = (CO_2_ or C_i_) × photophosphorylation**

**PLD* = ABA + NO**

**ABI1* = *Not* ABA**

**ROS* = (photophosphorylation>0) *And* PLD *And Not* ABI1**

AnionCh truth table:

An intermediate variable is calculated first:

**Anion_highactivation_ = (([Ca^2+^]_c_ = 2) *Or* ABA) *And Not* ABI1 *Or* (C_i_ = 2)**

| **Anion_highactivation_** | **phot1_complex_** | **Blue Light** | **AnionCh^*^** |
| --- | --- | --- | --- |
| 0 | 0 | 0 | 1 |
|  | 0 | 1 | 0 |
|  | 1 | Any | 0 |
| 1 | Any | Any | 1.6 |

**MCPS* = (Blue Light +Red Light) × (C_i_>0)**

SO truth table:

| **[K^+^]_v_** | **Sucrose** | **SO^*^** |
| --- | --- | --- |
| 0 | 0 | 0 |
|  | Sucrose >0 | 1 |
| 0< [K^+^]_v_ <=1 | Any | 1 |
| 1<[K^+^]_v_ <2 | Any | 2 |
| 2<=[K^+^]_v_ <6 | Any | 3 |
| 6<=[K^+^]_v_<9 | Any | 5 |
| 9<=[K^+^]_v_ | Any | 6 |

1. To distinguish from the subtraction operator ‘–‘, all dashes in the node names of this file are removed. Ca^2+^-ATPase is written as Ca^2+^ATPase, and H^+^ -ATPase_complex_ is written as H^+^ ATPase_complex_ [↑](#footnote-ref-1)
