# Supplementary material for: Analysis of a dynamic model of guard cell signaling reveals the stability of signal propagation
Source: BMC Syst Biol. 2016 Aug 19;10:78. doi: 10.1186/s12918-016-0327-7 (PMC4992220; doi:10.1186/s12918-016-0327-7)
Supplement: Additional file 2: — Compilation of comparisons between published experimental observations and the reduced model’s results for simulations of the identical conditions. (DOCX 163 kb) [file 12918_2016_327_MOESM2_ESM.docx]

Compilation of comparisons between published experimental observations and the reduced model's results for simulations of the identical conditions.

In the column entitled Simulation Settings “BL” stands for blue light, “RL” stands for red light, “ABA” means abscisic acid, “CO_2_” indicates atmospheric CO_2_, and “C_i_” indicates intercellular CO_2_. “Simulation Result” has two columns indicating the simulation from the Sun et al. model and the reduced model, respectively. All simulations of the reduced model are done for a sufficiently high number of time steps (1500) so that the system converges into an attractor. The initial conditions of all nodes are the same as in the Sun et al. model. Of all non-input nodes, only one is not zero in the initial condition: C_i_ =1, the others are assumed to start at 0. This is to represent their resting states before receiving a signal.

Some nodes have been reduced during model simplification by merging them with their sole regulators; two others were removed because they do not affect stomatal opening. In the sole case where the node measured in the experiment, AtrbohD/F, is reduced, we use the stabilized states of its upstream regulators to calculate the effective level of AtrbohD/F. In the sole case where the node measured in the experiment, [malate^2-^]_c_, was removed, we no longer consider this experimental observation. If the experiments express the manipulation of a node that was reduced by merging, we find the direct successors of the reduced node from the original model, re-evaluate their regulatory function with the perturbation plugged in, and use the result as a proxy of the manipulation. This approach has exactly the same effect as the manipulation of the node in the original model.

The last column is the qualitative evaluation of the consistency between the reduced model’s result and the relevant experimental observations. C stands for consistent, PC for partially consistent, and IC for inconsistent, with explanations added as necessary. The Boolean conversion is a map from the reduced model, thus the Boolean-converted reduced model has exactly the same results as the reduced model when interpreted. So we do not show the results of the Boolean-converted reduced model.

There are only three inconsistencies caused by simplification. Each can be fixed by adding a level of stomatal opening, respectively.

| **Experimental Observation** | **References** | **Simulation Settings** | **Simulation Result** | | **Consistency** |
| --- | --- | --- | --- | --- | --- |
|  |  |  | **Sun. et al model** | **Reduced model** |  |
| Under equal quantum flux, blue light is more efficient than red light in inducing stomatal opening. | [[1-3](#_ENREF_1)] | Blue light: BL=1, RL=0, ABA=0, CO_2_=C_i_=1 | SO=4.15 | SO=3 | C |
|  |  | Red light: BL=0, RL=1, ABA=0, CO_2_=C_i_=1 | SO=1 | SO=1 |  |
| Red background illumination synergistically increases the stomatal response to low intensity blue light. | [[4-6](#_ENREF_4)] | Monochromatic red light: BL=0, RL=1, ABA=0, CO_2_=C_i_=1 | SO=1 | SO=1 | C |
|  |  | Monochromatic blue light: BL=1, RL=0, ABA=0, CO_2_=C_i_=1 | SO=4.15 | SO=3 |  |
|  |  | Blue light with red light background: BL=1, RL=1, ABA=0, CO_2_=C_i_=1 | SO=11.28 | SO=5 |  |
| phot1 single knockout mutation does not inhibit blue light-induced stomatal opening. | [[7](#_ENREF_7)] | Wild type under blue light: BL=1, RL=0, ABA=0, CO_2_=C_i_=1 | SO=4.15 | SO=3 | C  (phot1 knockout is simulated by setting phot1_complex_=0) |
|  |  | phot1 knockout under blue light: BL=1, RL=0, ABA=0, CO_2_=C_i_=1, phot1 is kept 0 | SO=4.15 | SO=3 |  |
| phot2 single knockout mutation does not inhibit blue light-induced stomatal opening. | [[7](#_ENREF_7)] | Wild type under blue light: BL=1, RL=0, ABA=0, CO_2_=C_i_=1 | SO=4.15 | SO=3 | C  (phot2 knockout is simulated by modifying the regulatory functions of PLC, PLA2, PP1_cc_, ROP2, and AnionCh.) |
|  |  | phot2 knockout under blue light: BL=1, RL=0, ABA=0, CO_2_=C_i_=1, phot2 is kept 0 | SO=4.15 | SO=3 |  |
| phot1 and phot2 double knockout mutation inhibits blue light-induced stomatal opening. | [[7](#_ENREF_7)] | Wild type under blue light: BL=1, RL=0, ABA=0, CO_2_=Ci=1 | SO=4.15 | SO=3 | C  (This double knockout is simulated by modifying the regulatory functions of phot1_complex_, PLC, PLA2, PP1_cc_, ROP2, and AnionCh.) |
|  |  | phot1 and phot2 double knockout under blue light: BL=1, RL=0, ABA=0, CO_2_=C_i_=1, both phot1 and phot2 are kept 0 | SO=1 | SO=1 |  |
| phot1 and phot2 double knockout mutation does not inhibit red light-induced stomatal opening. | [[7](#_ENREF_7), [8](#_ENREF_8)] | Wild type under red light: BL=0, RL=1, ABA=0, CO_2_=C_i_=1 | SO=1 | SO=1 | C  (This double knockout is simulated by modifying the regulatory functions of phot1_complex_, PLC, PLA2, PP1_cc_, ROP2, and AnionCh.) |
|  |  | phot1 and phot2 double knockout under red light: BL=0, RL=1, ABA=0, CO_2_=C_i_=1, both phot1 and phot2 are kept 0 | SO=1 | SO=1 |  |
| phot1 and phot2 double knockout mutation inhibits white light-induced stomatal opening. | [[7](#_ENREF_7), [8](#_ENREF_8)] | Wild type under white light: BL=1, RL=1, ABA=0, CO_2_=C_i_=1 | SO=11.28 | 5 | C  (This double knockout is simulated by modifying the regulatory functions of phot1_complex_, PLC, PLA2, PP1_cc_, ROP2, and AnionCh.) |
|  |  | phot1 and phot2 double knockout under white light: BL=1, RL=1, ABA=0, CO_2_=C_i_=1, both phot1 and phot2 are kept 0 | SO=4.36 | 3 |  |
| Cytosolic Ca^2+^ oscillates in response to blue light. | [[9](#_ENREF_9)] | BL=1, RL=0, ABA=0, CO_2_=C_i_=1 | [Ca^2+^]_c_ oscillates between 0 and 1 | [Ca^2+^]_c_ oscillates between 0 and 1 | C |
| phot1 and phot2 double knockout reduces cytosolic Ca^2+^ response to blue light. | [[9](#_ENREF_9)] | Wild type under blue light: BL=1, RL=0, ABA=0, CO_2_=C_i_=1 | [Ca^2+^]_c_ oscillates between 0 and 1 | [Ca^2+^]_c_ oscillates between 0 and 1 | C  (This double knockout is simulated by modifying the regulatory functions of phot1_complex_, PLC, PLA2, PP1_cc_, ROP2, and AnionCh.) |
|  |  | phot1 and phot2 double knockout under blue light: BL=1, RL=0, ABA=0, CO_2_=C_i_=1, both phot1 and phot2 are kept 0 | [Ca^2+^]_c_=0 | [Ca^2+^]_c_=0 |  |
| Cytosolic Ca^2+^ does not respond to red light. | [[9](#_ENREF_9)] | BL=0, RL=1, ABA=0, CO_2_=C_i_=1 | [Ca^2+^]_c_=0 | [Ca^2+^]_c_=0 | C |
| Protein phosphatase inhibitors inhibit blue light-induced stomatal opening. | [[10](#_ENREF_10)] | Without protein phosphatase inhibitor under blue light: BL=1, RL=0, ABA=0, CO_2_=C_i_=1 | SO=4.15 | SO=3 | C |
|  |  | With protein phosphatase inhibitor under blue light: BL=1, RL=0, ABA=0, CO_2_=C_i_=1, PP1_cc_ is kept 0 | SO=1 | SO=1 |  |
| The protein phosphatase 1 inhibitor tautomycin inhibits white light-induced opening. | [[11](#_ENREF_11), [12](#_ENREF_12)] | Without protein phosphatase inhibitor under white light: BL=1, RL=1, ABA=0, CO_2_=C_i_=1 | SO=11.28 | SO=5 | C |
|  |  | With protein phosphatase inhibitor under white light: BL=1, RL=1, ABA=0, CO_2_=C_i_=1, PP1_cc_ is kept 0 | SO=2 | SO=1 |  |
| The protein phosphatase 1 inhibitor tautomycin does not inhibit red light-induced opening. | [[11](#_ENREF_11), [12](#_ENREF_12)] | Without protein phosphatase inhibitor under red light: BL=0, RL=1, ABA=0, CO_2_=C_i_=1 | SO=1 | SO=1 | C |
|  |  | With protein phosphatase inhibitor under red light: BL=0, RL=1, ABA=0, CO_2_=C_i_=1, PP1_cc_ is kept 0 | SO=1 | SO=1 |  |
| PRSL1 knockout mutation inhibits dual beam-induced stomatal opening. | [[12](#_ENREF_12)] | Wild type under dual beam: BL=1, RL=1, ABA=0, CO_2_=C_i_=1 | SO=11.28 | SO=5 | C  (PRSL1 knockout is simulated by modifying the regulatory function of PP1_cc_.) |
|  |  | PRSL1 knockout under dual beam: BL=1, RL=1, ABA=0, CO_2_=C_i_=1, PRSL1 is kept 0 | SO=4.36 | SO=3 |  |
| PRSL1 knockout does not inhibit red light-induced stomatal opening. | [[12](#_ENREF_12)] | Wild type under red light: BL=0, RL=1, ABA=0, CO_2_=C_i_=1 | SO=1 | SO=1 | C  (PRSL1 knockout is simulated by modifying the regulatory function of PP1_cc_.) |
|  |  | PRSL1 knockout under red light: BL=0, RL=1, ABA=0, CO_2_=C_i_=1, PRSL1 is kept 0 | SO=1 | SO=1 |  |
| Blue light activates the H^+^-ATPase. | [[13](#_ENREF_13), [14](#_ENREF_14)] | BL=1, RL=0, ABA=0, CO_2_=C_i_=1 | H^+^-ATPase_complex_ =2 | H^+^-ATPase_complex_ =2 | C |
| phot1 and phot2 double knockout mutation inhibits blue light-activated H^+^-ATPase activity. | [[13](#_ENREF_13)] | Wild type under blue light: BL=1, RL=0, ABA=0, CO_2_=C_i_=1 | H^+^-ATPase_complex_ =2 | H^+^-ATPase_complex_ =2 | C  (This double knockout is simulated by modifying the regulatory functions of phot1_complex_, PLC, PLA2, PP1_cc_, ROP2, and AnionCh.) |
|  |  | phot1 and phot2 double knockout under blue light: BL=1, RL=0, ABA=0, CO_2_=C_i_=1, both phot1 and phot2 are kept 0 | H^+^-ATPase_complex_ =0 | H^+^-ATPase_complex_ =0 |  |
| Red light does not activate the H^+^-ATPase. | [[13](#_ENREF_13), [14](#_ENREF_14)] | BL=0, RL=1, ABA=0, CO_2_=C_i_=1 | H^+^-ATPase_complex_ =0 | H^+^-ATPase_complex_ =0 | C |
| Red light does not enhance blue light-dependent H^+^-ATPase activity under a condition of excess ATP and fixed C_i_. | [[14](#_ENREF_14)] | Blue light with excess ATP and fixed C_i_: BL=1, RL=0, ABA=0, ATP=3, CO_2_ and C_i_ are fixed at a certain value, e.g. CO_2_=C_i_=1 | H^+^-ATPase_complex_ =3 | H^+^-ATPase_complex_ =3 | C |
|  |  | Blue light and red light with excess ATP and fixed C_i_: BL=1, RL=1, ABA=0, ATP=3, CO_2_ and C_i_ are fixed at the same value as they are in the previous condition | H^+^-ATPase_complex_  =3 | H^+^-ATPase_complex_  =3 |  |
| Inhibiting the H^+^-ATPase with vanadate inhibits white light-induced stomatal opening. | [[15](#_ENREF_15), [16](#_ENREF_16)] | Without vanadate under white light: BL=1, RL=1, ABA=0, CO_2_=C_i_=1 | SO=11.28 | SO=5 | C |
|  |  | With vanadate under white light: BL=1, RL=1, ABA=0, CO_2_=C_i_=1, H^+^-ATPase_complex_ is kept 0 | SO=2 | SO=1 |  |
| Fusicoccin stimulates stomatal opening. | [[17](#_ENREF_17)] | Dark without fusicoccin: BL=0, RL=0, ABA=0, CO_2_=C_i_=1 | SO=0 | SO=0 | C |
|  |  | Dark with fusicoccin: BL=0, RL=0, ABA=0, CO_2_=C_i_=1, H^+^-ATPase_complex_ is kept 9 | SO=14.18 | SO=6 |  |
| Fusicoccin stimulates guard cell K^+^ uptake. | [[17](#_ENREF_17)] | Dark without fusicoccin: BL=0, RL=0, ABA=0, CO_2_=C_i_=1 | [K^+^]_c_=0 | [K^+^]_c_ =0 | C |
|  |  | Dark with fusicoccin: BL=0, RL=0, ABA=0, CO_2_=C_i_=1, H^+^-ATPase_complex_ is kept 9 | [K^+^]_c_=9 | [K^+^]_c_ =9 |  |
| CO_2_-free air promotes white light-induced stomatal opening. | [[18](#_ENREF_18)] | White light in ambient air: BL=1, RL=1, ABA=0, CO_2_=C_i_=1 | SO=11.28 | SO=5 | C |
|  |  | White light in CO_2_-free air: BL=1, RL=1, ABA=0, CO_2_=C_i_=0 | SO==14.01 | SO=6 |  |
| Reduced CO_2_ concentration enhances red light-induced stomatal opening. | [[19](#_ENREF_19)] | Red light in ambient air: BL=0, RL=1, ABA=0, CO_2_=C_i_=1 | SO=1 | SO=1 | C |
|  |  | Red light in reduced CO_2_ air: BL=0, RL=1, ABA=0, CO_2_=C_i_=0 | SO=3.15 | SO=3 |  |
| Reduced CO_2_ concentration enhances blue light-induced stomatal opening. | [[4](#_ENREF_4)] | Blue light in ambient air: BL=1, RL=0, ABA=0, CO_2_=C_i_=1 | SO=4.15 | SO=3 | C |
|  |  | Blue light in reduced CO_2_ air: BL=1, RL=0, ABA=0, CO_2_=C_i_=0 | SO=9.28 | SO=5 |  |
| The plasma membrane hyperpolarizes under red light in CO_2_-free air. | [[20](#_ENREF_20)] | BL=0, RL=1, ABA=0, CO_2_=C_i_=0 | PMV=-1 | PMV=-1 | C |
| High CO_2_ inhibits stomatal opening. | [[21](#_ENREF_21)] | Moderate CO_2_: BL=1, RL=1, ABA=0, CO_2_=C_i_=1 | SO=11.28 | SO=5 | C |
|  |  | High CO_2_: BL=1, RL=1, ABA=0, CO_2_=C_i_=2 | SO=2 | SO=1 |  |
| High C_i_ depolarizes the plasma membrane. | [[22](#_ENREF_22)] | Moderate CO_2_: BL=1, RL=1, ABA=0, CO_2_=C_i_=1 | PMV=-2 | PMV=-2 | C |
|  |  | High CO_2_: BL=1, RL=1, ABA=0, CO_2_=C_i_=2 | PMV=0 | PMV=0 |  |
| The guard cell plasma membrane depolarizes under red light in CO_2_-containing air. | [[20](#_ENREF_20)] | BL=0, RL=1, ABA=0, CO_2_=C_i_=1 | PMV=0 | PMV=0 | C |
| The plasma membrane hyperpolarizes in response to light, and depolarizes in the dark. | [[23](#_ENREF_23)] | Light: BL=1, RL=1, ABA=0, CO_2_=C_i_=1 | PMV=-2 | PMV=-2 | C |
|  |  | Dark: BL=0, RL=0, ABA=0, CO_2_=C_i_=1 | PMV=0 | PMV=0 |  |
| Under equal quantum flux, blue light is more efficient than red light in inducing Rb^+^ (a K^+^ equivalent) uptake. | [[1](#_ENREF_1), [2](#_ENREF_2)] | Blue light: BL=1, RL=0, ABA=0, CO_2_=C_i_=1 | [K^+^]_c_=2 | [K^+^]_c_=2 | C |
|  |  | Red light: BL=0, RL=1, ABA=0, CO_2_=C_i_=1 | [K^+^]_c_=0 | [K^+^]_c_=0 |  |
| White light-induced stomatal opening is inhibited by K_in_ channel knockout mutation. | [[24](#_ENREF_24)] | Wild type under white light: BL=1, RL=1, ABA=0, CO_2_=C_i_=1 | SO=11.28 | SO=5 | C |
|  |  | K_in_ channel knockout under white light: BL=1, RL=1, ABA=0, CO_2_=C_i_=1, K_in_ is kept 0 | SO=2 | SO=1 |  |
| Blue light-induced stomatal opening is inhibited by K_in_ channel knockout mutation. | [[24](#_ENREF_24)] | Wild type under blue light: BL=1, RL=0, ABA=0, CO_2_=C_i_=1 | SO=4.15 | SO=3 | C |
|  |  | K_in_ channel knockout under blue light: BL=1, RL=0, ABA=0, CO_2_=C_i_=1, K_in_ is kept 0 | SO=1 | SO=1 |  |
| Red light-induced stomatal opening is not inhibited by K_in_ channel knockout mutation. | [[24](#_ENREF_24)] | Wild type under red light: BL=0, RL=1, ABA=0, CO_2_=C_i_=1 | SO=1 | SO=1 | C |
|  |  | K_in_ channel knockout under red light: BL=0, RL=1, ABA=0, CO_2_=C_i_=1, K_in_ is kept 0 | SO=1 | SO=1 |  |
| Nitrate transporter CHL1 knockout inhibits white light-induced stomatal opening. | [[25](#_ENREF_25)] | Wild type under white light: BL=1, RL=1, ABA=0, CO_2_=C_i_=1 | SO=11.28 | SO=5 | IC  (due to the elimination of anions from the SO regulatory function) |
|  |  | CHL1 knockout under white light: BL=1, RL=1, ABA=0, CO_2_=C_i_=1, CHL1 is kept 0 | SO=10.68 | SO=5 |  |
| The rate of malate formation under blue light with a red light background is larger than the sum of rates under monochromatic blue or red light. | [[26](#_ENREF_26)] | Monochromatic red light: BL=0, RL=1, ABA=0, CO_2_=C_i_=1 | [malate^2-^]_c_=0 | Reduced | Cannot be captured (due to the elimination of anions from the SO regulatory function) |
|  |  | Monochromatic blue light: BL=1, RL=0, ABA=0, CO_2_=C_i_=1 | [malate^2-^]_c_=1.5 | Reduced |  |
|  |  | Blue light with a red light background: BL=1, RL=1, ABA=0, CO_2_=C_i_=1 | [malate^2-^]_c_=6.5 | Reduced |  |
| Malate transporter AtABCB14 knockout mutant displays reduced white light-induced stomatal opening. | [[27](#_ENREF_27)] | Wild type under white light: BL=1, RL=1, ABA=0, CO_2_=C_i_=1 | SO=11.28 | SO=5 | IC  (Original model is IC) |
|  |  | AtABCB14 knockout under white light: BL=1, RL=1, ABA=0, CO_2_=C_i_=1, AtABCB14 is kept 0 | SO=11.28 | SO=5 |  |
| Sucrose concentration increases during white light-induced stomatal opening. | [[28](#_ENREF_28)] | Dark: BL=0, RL=0, ABA=0, CO_2_=C_i_=1 | Sucrose=0 | Sucrose=0 | C |
|  |  | White light: BL=1, RL=1, ABA=0, CO_2_=C_i_=1 | Sucrose=2 | Sucrose=2 |  |
| Sucrose concentration increases during blue light-induced stomatal opening. | [[29](#_ENREF_29)] | Dark: BL=0, RL=0, ABA=0, CO_2_=C_i_=1 | Sucrose=0 | Sucrose=0 | C |
|  |  | Blue light: BL=1, RL=0, ABA=0, CO_2_=C_i_=1 | Sucrose=1 | Sucrose=1 |  |
| Sucrose concentration increases during red light-induced stomatal opening. | [[29](#_ENREF_29)] | Dark: BL=0, RL=0, ABA=0, CO_2_=C_i_=1 | Sucrose=0 | Sucrose=0 | C |
|  |  | Red light: BL=0, RL=1, ABA=0, CO_2_=C_i_=1 | Sucrose=1 | Sucrose=1 |  |
| PLA_2_β knockout mutant exhibits reduced white light-induced stomatal opening compared to wild type. | [[30](#_ENREF_30)] | Wild type under white light: BL=1, RL=1, ABA=0, CO_2_=C_i_=1 | SO=11.28 | SO=5 | C |
|  |  | PLA_2_β knockout under white light: BL=1, RL=1, ABA=0, CO_2_=C_i_=1, PLA_2_β is kept 0 | SO=2 | SO=1 |  |
| PIP_2_ knockout mutant displays reduced white light-induced stomatal opening compared to wild type. | [[31](#_ENREF_31)] | Wild type under white light: BL=1, RL=1, ABA=0, CO_2_=C_i_=1 | SO=11.28 | SO=5 | C  (PIP2_PM_ knockout is simulated by modifying the regulatory function of AnionCh.) |
|  |  | PIP_2_ knockout under white light: BL=1, RL=1, ABA=0, CO_2_=C_i_=1, PIP2_PM_ is kept 0 | SO=2 | SO=1 |  |
| Dominant negative mutant of the small G protein ROP2 exhibits enhanced stomatal opening in response to white light. | [[32](#_ENREF_32)] | Wild type under white light: BL=1, RL=1, ABA=0, CO_2_=C_i_=1 | SO=11.28 | SO=5 | IC  (Due to the elimination of ROP2- RIC7 from the SO regulatory function) |
|  |  | Dominant negative small G protein ROP2 mutant under white light: BL=1, RL=1, ABA=0, CO_2_=C_i_=1, ROP2 is kept 0 | Stomatal opening=11.45 | SO=5 |  |
| ABA inhibits white light-induced stomatal opening. | [[13](#_ENREF_13), [33-36](#_ENREF_33)] | White light without ABA: BL=1, RL=1, ABA=0, CO_2_=C_i_=1 | SO=11.28 | SO=5 | C |
|  |  | White light with ABA: BL=1, RL=1, ABA=1, CO_2_=C_i_=1 | SO=0 | SO=0 |  |
| ABA inhibits blue light-induced stomatal opening. | [[37](#_ENREF_37)] | Blue light without ABA: BL=1, RL=0, ABA=0, CO_2_=C_i_=1 | SO=4.15 | SO=3 | C |
|  |  | Blue light with ABA: BL=1, RL=0, ABA=1, CO_2_=C_i_=1 | SO=0 | SO=0 |  |
| ABA induces cytosolic Ca^2+^ oscillation. | [[35](#_ENREF_35)] | BL=1, RL=1, ABA=1, CO_2_=C_i_=1 | In the model cytosolic Ca^2+^ increases, peaks, then it decreases in response to ABA; there is no consecutive increase. | In the model cytosolic Ca^2+^ increases, peaks, then it decreases in response to ABA; there is no consecutive increase. | PC  (Original model is PC) |
| ROS inhibits white light-induced stomatal opening. | [[38](#_ENREF_38)] | White light without ROS: BL=1, RL=1, ABA=0, CO_2_=C_i_=1 | SO=11.28 | SO=5 | C |
|  |  | White light with ROS: BL=1, RL=1, ABA=0, CO_2_=C_i_=1, ROS is kept 1 | SO=8.92 | SO=3 |  |
| ROS inhibits blue light-induced stomatal opening. | [[37](#_ENREF_37)] | Blue light without ROS: BL=1, RL=0, ABA=0, CO_2_=C_i_=1 | SO=4.15 | SO=3 | C |
|  |  | Blue light with ROS: BL=1, RL=0, ABA=0, CO_2_=C_i_=1, ROS is kept 1 | Stomatal opening=3.84 | SO=2 |  |
| NO donor SNP inhibits white light-induced stomatal opening. | [[38](#_ENREF_38)] | White light without NO donor SNP: BL=1, RL=1, ABA=0, CO_2_=C_i_=1 | SO=11.28 | SO=5 | C |
|  |  | White light with NO donor SNP: BL=1, RL=1, ABA=0, CO_2_=C_i_=1, NO is kept 1 | SO=8.92 | SO=3 |  |
| NO donor SNP inhibits blue light-induced stomatal opening. | [[37](#_ENREF_37), [39](#_ENREF_39)] | Blue light without NO donor SNP: BL=1, RL=0, ABA=0, CO_2_=C_i_=1 | SO=4.15 | SO=3 | C |
|  |  | Blue light with NO donor SNP: BL=1, RL=0, ABA=0, CO_2_=C_i_=1, NO is kept 1 | SO=3.84 | SO=2 |  |
| NO donor SNP does not inhibit red light-induced stomatal opening. | [[39](#_ENREF_39)] | Red light without NO donor SNP: BL=0, RL=1, ABA=0, CO_2_=C_i_=1 | SO=1 | SO=1 | C |
|  |  | Red light with NO donor SNP: BL=0, RL=1, ABA=0, CO_2_=C_i_=1, NO is kept 1 | SO=1 | SO=1 |  |
| NO scavenger PTIO partially restores stomatal opening inhibited by ABA. | [[40](#_ENREF_40)] | White light without ABA: BL=1, RL=1, ABA=0, CO_2_=C_i_=1 | SO=11.28 | SO=5 | C |
|  |  | White light with ABA: BL=1, RL=1, ABA=1, CO_2_=C_i_=1 | SO=0 | SO=0 |  |
|  |  | White light with ABA and NO scavenger PTIO: BL=1, RL=1, ABA=1, CO_2_=C_i_=1, NO is kept 0 | SO=5.18 | SO=3 |  |
| Anion channel blocker 9-AC reverses inhibition of white light-induced stomatal opening by ABA. | [[34](#_ENREF_34)] | White light without ABA: BL=1, RL=1, ABA=0, CO_2_=C_i_=1 | SO=11.28 | SO=5 | C |
|  |  | White light with ABA: BL=1, RL=1, ABA=1, CO_2_=C_i_=1 | SO=0 | SO=0 |  |
|  |  | White light with ABA and anion channel blocked by 9-AC: BL=1, RL=1, ABA=1, CO_2_=C_i_=1, AnionCh is kept 0 | SO=1.73 | SO=2 |  |
| ABA can activate anion efflux channels without the mediation of Ca^2+^. | [[41](#_ENREF_41)] | With the mediation of Ca^2+^: BL=1, RL=1, ABA=1, CO_2_=C_i_=1 | AnionCh=1.6 | AnionCh=1.6 | C |
|  |  | Without the mediation of Ca^2+^: BL=1, RL=1, ABA=1, CO_2_=C_i_=1, [Ca^2+^]_c_ is kept 0 | AnionCh=1.6 | AnionCh=1.6 |  |
| ABA inhibits blue light-induced H^+^-ATPase activity. | [[42](#_ENREF_42)] | Without ABA: BL=1, RL=0, ABA=0, CO_2_=C_i_=1 | H^+^-ATPase_complex_=2 | H^+^-ATPase_complex_=2 | C |
|  |  | With ABA: BL=1, RL=0, ABA=1, CO_2_=C_i_=1 | H^+^-ATPase_complex_=1 | H^+^-ATPase_complex_=1 |  |
| ROS inhibits blue light-induced H^+^-ATPase activity. | [[42](#_ENREF_42)] | Without ROS: BL=1, RL=0, ABA=0, CO_2_=C_i_=1 | H^+^-ATPase_complex_=2 | H^+^-ATPase_complex_=2 | C |
|  |  | With ROS: BL=1, RL=0, ABA=0, CO_2_=C_i_=1, ROS is kept 1 | H^+^-ATPase_complex_=1.8 | H^+^-ATPase_complex_=1.8 |  |
| ROS scavenger partially restores blue light-dependent H^+^-ATPase activity inhibited by ABA. | [[42](#_ENREF_42)] | Blue light without ABA: BL=1, RL=0, ABA=0, CO_2_=C_i_=1 | H^+^-ATPase_complex_=2 | H^+^-ATPase_complex_=2 | C |
|  |  | Blue light with ABA: BL=1, RL=0, ABA=1, CO_2_=C_i_=1 | H^+^-ATPase_complex_=1 | H^+^-ATPase_complex_=1 |  |
|  |  | Blue light with ABA and ROS scavenger: BL=1, RL=0, ABA=1, CO_2_=C_i_=1, ROS is kept 0 | H^+^-ATPase_complex_=1.8 | H^+^-ATPase_complex_=1.8 |  |
| PA inhibits white light-induced stomatal opening. | [[36](#_ENREF_36), [43](#_ENREF_43)] | White light: BL=1, RL=1, ABA=0, CO_2_=C_i_=1 | SO=11.28 | SO=5 | C  (PA=1 perturbation is simulated by setting PLD=1.) |
|  |  | White light with sustained PA: BL=1, RL=1, ABA=0, CO_2_=C_i_=1, PA is kept 1 | SO=8.92 | SO=3 |  |
| PA inhibits blue light-induced stomatal opening. | [[37](#_ENREF_37)] | Blue light: BL=1, RL=0, ABA=0, CO_2_=C_i_=1 | SO=4.15 | SO=3 | C  (PA=1 perturbation is simulated by setting PLD=1.) |
|  |  | Blue light with sustained PA: BL=1, RL=0, ABA=0, CO_2_=C_i_=1, PA is kept 1 | SO=3.84 | SO=2 |  |
| PA does not inhibit red light-induced stomatal opening. | [[37](#_ENREF_37)] | Red light: BL=0, RL=1, ABA=0, CO_2_=C_i_=1 | SO=1 | SO=1 | C  (PA=1 perturbation is simulated by setting PLD=1.) |
|  |  | Red light with sustained PA: BL=0, RL=1, ABA=0, CO_2_=C_i_=1, PA is kept 1 | SO=1 | SO=1 |  |
| The inhibition (with 1-buOH) of PA production elicited by ABA partially prevents ABA's inhibition of white light-induced stomatal opening. | [[43](#_ENREF_43)] | White light without ABA: BL=1, RL=1, ABA=0, CO_2_=C_i_=1 | SO=11.28 | SO=5 | IC  (due to the elimination of anions from the SO regulatory function) |
|  |  | White light with ABA: BL=1, RL=1, ABA=1, CO_2_=C_i_=1 | SO=0 | SO=0 |  |
|  |  | White light with ABA and PA inhibitor 1-buOH: BL=1, RL=1, ABA=1, CO_2_=C_i_=1, PA is kept 0 | SO=6.9 | SO=5 |  |
| OST1 knockout mutation does not affect light-induced stomatal opening. | [[13](#_ENREF_13), [44](#_ENREF_44)] | Wild type under light: BL=1, RL=1, ABA=0, CO_2_=C_i_=1 | SO=11.28 | SO=5 | C |
|  |  | OST1 knockout mutant under light: BL=1, RL=1, ABA=0, CO_2_=C_i_=1, OST1 is kept 0 | SO=11.28 | SO=5 |  |
| OST1 knockout mutation disrupts ABA's inhibition of white light-induced stomatal opening. | [[13](#_ENREF_13), [44](#_ENREF_44)] | Wild type under white light without ABA: BL=1, RL=1, ABA=0, CO_2_=C_i_=1 | SO=11.28 | SO=5 | C  (OST1 perturbation is simulated by modifying the regulatory function of ROS.) |
|  |  | Wild type under white light with ABA: BL=1, RL=1, ABA=1, CO_2_=C_i_=1 | SO=0 | SO=0 |  |
|  |  | OST1 knockout mutant under white light with ABA: BL=1, RL=1, ABA=1, CO_2_=C_i_=1, OST1 is kept 0 | SO=5.18 | SO=3 |  |
| ABA upregulates NADPH oxidases AtrbohD/F. | [[45](#_ENREF_45)] | Without ABA: BL=1, RL=1, ABA=0, CO_2_=C_i_=1 | AtrbohD/F=0 | AtrbohD/F=0 | C  (Although reduced, AtrbohD/F is evaluated using the stabilized states of its regulators PLD and ABI1.) |
|  |  | With ABA: BL=1, RL=1, ABA=1, CO_2_=C_i_=1 | AtrbohD/F=1 | AtrbohD/F=1 |  |
| AtrbohD/F double knockout mutation impairs ROS production in response to ABA compared to wild type. | [[45](#_ENREF_45)] | Wild type with ABA: BL=1, RL=1, ABA=1, CO_2_=C_i_=1 | ROS=1 | ROS=1 | C  (AtrbohD/F double knockout is simulated by modifying the regulatory function of ROS.) |
|  |  | AtrbohD/F double knockout mutant with ABA: BL=1, RL=1, ABA=1, CO_2_=C_i_=1, AtrbohD/F is kept 0 | ROS=0 | ROS=0 |  |
| Inhibiting NADPH oxidase with DPI partially restores stomatal opening inhibited by ABA. | [[46](#_ENREF_46)] | White light without ABA: BL=1, RL=1, ABA=0, CO_2_=C_i_=1; | SO=11.28 | SO=5 | C  (AtrbohD/F double knockout is simulated by modifying the regulatory function of ROS.) |
|  |  | White light with ABA: BL=1, RL=1, ABA=1, CO_2_=C_i_=1 | SO=0 | SO=0 |  |
|  |  | White light with ABA and NADPH oxidase inhibitor DPI: BL=1, RL=1, ABA=1, CO_2_=C_i_=1, AtrbohD/F is kept 0 | SO=5.18 | SO=3 |  |

**References:**

1. Tallman, G. and E. Zeiger, *Light quality and osmoregulation in vicia guard cells : evidence for involvement of three metabolic pathways.* Plant Physiol, 1988. **88**(3): p. 887-95.

2. Hsiao, T.C. and W.G. Allaway, *Action Spectra for Guard Cell Rb Uptake and Stomatal Opening in Vivia faba.* Plant Physiol, 1973. **51**(1): p. 82-8.

3. Sharkey, T.D. and K. Raschke, *Effect of Light Quality on Stomatal Opening in Leaves of Xanthium strumarium L.* Plant Physiol, 1981. **68**(5): p. 1170-4.

4. Assmann, S.M., *Enhancement of the Stomatal Response to Blue Light by Red Light, Reduced Intercellular Concentrations of CO(2), and Low Vapor Pressure Differences.* Plant Physiol, 1988. **87**(1): p. 226-31.

5. Shimazaki, K., et al., *Light regulation of stomatal movement.* Annu Rev Plant Biol, 2007. **58**: p. 219-47.

6. Karlsson, P.E., *Blue light regulation of stomata in wheat seedlings. I. Influence of red background illumination and initial conductance level.* Physiologia Plantarum, 1986. **66**: p. 5.

7. Kinoshita, T., et al., *Phot1 and phot2 mediate blue light regulation of stomatal opening.* Nature, 2001. **414**(6864): p. 656-60.

8. Boccalandro, H.E., et al., *Phototropins but not cryptochromes mediate the blue light-specific promotion of stomatal conductance, while both enhance photosynthesis and transpiration under full sunlight.* Plant Physiol, 2012. **158**(3): p. 1475-84.

9. Baum, G., et al., *Stimulation of the blue light phototropic receptor NPH1 causes a transient increase in cytosolic Ca2+.* Proc Natl Acad Sci U S A, 1999. **96**(23): p. 13554-9.

10. Kinoshita, T. and K. Shimazaki, *Involvement of calyculin A- and okadaic acid-sensitive protein phosphatase in the blue light response of stomatal guard cells.* Plant Cell Physiol, 1997. **38**: p. 5.

11. Takemiya, A., et al., *Protein phosphatase 1 positively regulates stomatal opening in response to blue light in Vicia faba.* Proc Natl Acad Sci U S A, 2006. **103**(36): p. 13549-54.

12. Takemiya, A., et al., *Identification of a regulatory subunit of protein phosphatase 1 which mediates blue light signaling for stomatal opening.* Plant Cell Physiol, 2013. **54**(1): p. 24-35.

13. Hayashi, M., et al., *Immunohistochemical detection of blue light-induced phosphorylation of the plasma membrane H+-ATPase in stomatal guard cells.* Plant Cell Physiol, 2011. **52**(7): p. 1238-48.

14. Taylor, A.R. and S.M. Assmann, *Apparent absence of a redox requirement for blue light activation of pump current in broad bean guard cells.* Plant Physiol, 2001. **125**(1): p. 329-38.

15. Schwartz, A., N. Illan, and S.M. Assmann, *Vanadate inhibition of stomatal opening in epidermal peels of Commelina communis : Cl(-) interferes with vanadate uptake.* Planta, 1991. **183**(4): p. 590-6.

16. Amodeo, G., A. Srivastava, and E. Zeiger, *Vanadate inhibits blue light-stimulated swelling of vicia guard cell protoplasts.* Plant Physiol, 1992. **100**(3): p. 1567-70.

17. Squire, G.R. and T.A. Mansfield, *The action of fusicoccin on stomatal guard cells and the subsidiary cells.* New Phytologist, 1974. **73**: p. 8.

18. Allaway, W.G. and T.A. Mansfield, *Stomatal Responses to Changes in Carbon Dioxide Concentration in Leaves Treated with 3-(4-Chlorophenyl)-I, I-Dimethylurea.* New Phytologist, 1967. **66**: p. 7.

19. Olsen, R.L., et al., *Red light activates a chloroplast-dependent ion uptake mechanism for stomatal opening under reduced CO2 concentrations in Vicia spp.* New Phytologist, 2002. **153**: p. 12.

20. Roelfsema, M.R., et al., *CO2 provides an intermediate link in the red light response of guard cells.* Plant J, 2002. **32**(1): p. 65-75.

21. Brearley, J., M.A. Venis, and M.R. Blatt, *The effect of elevated CO2 concentrations on K+ and anion channels of Vicia faba L. guard cells.* Planta, 1997. **203**: p. 10.

22. Edwards, A. and D.J.F. Bowling, *Evidence for a CO2 inhibited proton extrusion pump in the stomatal cells of Tradescantia virginiana.* Journal of Experimental Botany, 1985. **36**: p. 8.

23. Roelfsema, M.R., et al., *Single guard cell recordings in intact plants: light-induced hyperpolarization of the plasma membrane.* Plant J, 2001. **26**(1): p. 1-13.

24. Lebaudy, A., et al., *Plant adaptation to fluctuating environment and biomass production are strongly dependent on guard cell potassium channels.* Proc Natl Acad Sci U S A, 2008. **105**(13): p. 5271-6.

25. Guo, F.Q., J. Young, and N.M. Crawford, *The nitrate transporter AtNRT1.1 (CHL1) functions in stomatal opening and contributes to drought susceptibility in Arabidopsis.* Plant Cell, 2003. **15**(1): p. 107-17.

26. Ogawa, T., et al., *Synergistic action of red and blue light and action spectra for malate formation in guard cells of Vicia faba L.* Planta, 1978. **142**(1): p. 61-5.

27. Lee, M., et al., *The ABC transporter AtABCB14 is a malate importer and modulates stomatal response to CO2.* Nat Cell Biol, 2008. **10**(10): p. 1217-23.

28. Reddy, A.R. and V.S.R. Das, *Stomatal movements and sucrose uptake by guard cell protoplasts of Commelina benghalensis L.* Plant Cell Physiol, 1986. **27**: p. 6.

29. Talbott, L.D. and E. Zeiger, *Sugar and Organic Acid Accumulation in Guard Cells of Vicia faba in Response to Red and Blue Light.* Plant Physiol, 1993. **102**(4): p. 1163-1169.

30. Seo, J., et al., *Phospholipase A2beta mediates light-induced stomatal opening in Arabidopsis.* J Exp Bot, 2008. **59**(13): p. 3587-94.

31. Lee, Y., et al., *Phosphatidylinositol 4,5-bisphosphate is important for stomatal opening.* Plant J, 2007. **52**(5): p. 803-16.

32. Jeon, B.W., et al., *The Arabidopsis small G protein ROP2 is activated by light in guard cells and inhibits light-induced stomatal opening.* Plant Cell, 2008. **20**(1): p. 75-87.

33. Gepstein, S., M. Jacobs, and L. Taiz, *Inhibition of stomatal opening in Vicia faba epidermal tissue by vanadate and abscisic acid.* Plant Science Letters, 1982. **28**: p. 10.

34. Schwartz, A., et al., *Anion-Channel Blockers Inhibit S-Type Anion Channels and Abscisic Acid Responses in Guard Cells.* Plant Physiol, 1995. **109**(2): p. 651-658.

35. Staxen, I., et al., *Abscisic acid induces oscillations in guard-cell cytosolic free calcium that involve phosphoinositide-specific phospholipase C.* Proc Natl Acad Sci U S A, 1999. **96**(4): p. 1779-84.

36. Zhang, W., et al., *Phospholipase D alpha 1-derived phosphatidic acid interacts with ABI1 phosphatase 2C and regulates abscisic acid signaling.* Proc Natl Acad Sci U S A, 2004. **101**(25): p. 9508-13.

37. Takemiya, A. and K. Shimazaki, *Phosphatidic acid inhibits blue light-induced stomatal opening via inhibition of protein phosphatase 1 [corrected].* Plant Physiol, 2010. **153**(4): p. 1555-62.

38. Desikan, R., et al., *A new role for an old enzyme: nitrate reductase-mediated nitric oxide generation is required for abscisic acid-induced stomatal closure in Arabidopsis thaliana.* Proc Natl Acad Sci U S A, 2002. **99**(25): p. 16314-8.

39. Zhang, X., et al., *Nitric oxide inhibits blue light-specific stomatal opening via abscisic acid signaling pathways in Vicia guard cells.* Plant Cell Physiol, 2007. **48**(5): p. 715-23.

40. Neill, S., et al., *Nitric oxide, stomatal closure, and abiotic stress.* J Exp Bot, 2008. **59**(2): p. 165-76.

41. Levchenko, V., et al., *Cytosolic abscisic acid activates guard cell anion channels without preceding Ca2+ signals.* Proc Natl Acad Sci U S A, 2005. **102**(11): p. 4203-8.

42. Zhang, X., et al., *Inhibition of blue light-dependent H+ pumping by abscisic acid through hydrogen peroxide-induced dephosphorylation of the plasma membrane H+-ATPase in guard cell protoplasts.* Plant Physiol, 2004. **136**(4): p. 4150-8.

43. Jacob, T., et al., *Abscisic acid signal transduction in guard cells is mediated by phospholipase D activity.* Proc Natl Acad Sci U S A, 1999. **96**(21): p. 12192-7.

44. Mustilli, A.C., et al., *Arabidopsis OST1 protein kinase mediates the regulation of stomatal aperture by abscisic acid and acts upstream of reactive oxygen species production.* Plant Cell, 2002. **14**(12): p. 3089-99.

45. Kwak, J.M., et al., *NADPH oxidase AtrbohD and AtrbohF genes function in ROS-dependent ABA signaling in Arabidopsis.* EMBO J, 2003. **22**(11): p. 2623-33.

46. Pei, Z.M., et al., *Calcium channels activated by hydrogen peroxide mediate abscisic acid signalling in guard cells.* Nature, 2000. **406**(6797): p. 731-4.
