# Supplementary material for: Analysis of a dynamic model of guard cell signaling reveals the stability of signal propagation
Source: BMC Syst Biol. 2016 Aug 19;10:78. doi: 10.1186/s12918-016-0327-7 (PMC4992220; doi:10.1186/s12918-016-0327-7)
Supplement: Additional file 3: — Analysis of stomatal opening model. Detailed derivation of attractor analysis and other statements made in the main article. (DOCX 98 kb) [file 12918_2016_327_MOESM3_ESM.docx]

Theoretical Analysis of the Stomatal Opening Model

1. **Background:**

Let’s consider a discrete dynamic system where $x$is a state of the system, with $x_{k}$being the state of node k. Let $f_{k}\left( x \right)$be the regulatory function for node *k*. For all node pairs *i*, *j* there is a positive (negative) edge from *j* to *i* if there exists $x$and $x_{j}$ such that$x_{j}+1$ is an allowed state and $\left( f_{i}\left( x_{1},\ldots,x_{j}+1,\ldots,x_{n} \right)-f_{i}\left( x_{1},\ldots,x_{j},\ldots,x_{n} \right) \right)$ is positive (negative) [[1-3](#_ENREF_1)]. The set of edges defined this way can be called the interaction graph of the system. A circuit in the interaction graph is negative if it has an odd number of negative edges and is positive if it has no or an even number of negative edges.

Using this definition of edges, A. Richard [[1](#_ENREF_1), [2](#_ENREF_2)] proved two conjectures originally formulated by R. Thomas in the discrete case [[4](#_ENREF_4)]:

1. The presence of a negative circuit in the interaction graph of a discrete dynamical system is a necessary, but not sufficient, condition for the presence of sustained oscillations in the dynamics of the network.

2. The presence of a positive circuit is a necessary, but not sufficient, condition for the occurrence of multiple stable steady states for the system.

These conjectures can be directly applied to our model because the regulatory functions in the model satisfy the definition of edge signs used by A. Richard. A consequence of R. Thomas’ conjectures is that any node that is not part of a strongly connected component (SCC) cannot oscillate or have multiple stable steady states in the long-term behavior of a discrete dynamic system. This is true not only for the unperturbed system, but also in case of sustained node perturbations such as knockouts. The reason is that node perturbations cannot create new edges, so a node originally not in an SCC cannot become part of an SCC after any perturbation. Both the Sun et al. model and our reduced model assumes constant input signals and sustained perturbations, i.e. all input nodes or perturbed nodes will stay in a fixed state. We do not consider alternating signals for input nodes or perturbed nodes.

We analyze the three SCCs in the network: C_i_ SCC, NO Cycle, and Ion SCC. We use the Sun et al. model [[5](#_ENREF_5)] for the analysis of the C_i_ SCC.

1. **The C_i_ SCC does not admit oscillations or multi-stability under any perturbation**

The C_i_ SCC has three nodes, C_i_, mesophyll cell photosynthesis (MCPS), carbon fixation, and four edges that form two negative feedback loops, one between carbon fixation and C_i_, and the other between C_i_ and MCPS. Being composed of negative cycles, this SCC does not satisfy the necessary condition for multi-stability but it does satisfy the necessary condition for oscillations. To further evaluate the possibility of oscillations, we look at the specific regulatory functions of each node.

**carbon fixation^*^ = ((CO_2_> 0) *Or* (C_i_> 0)) × photophosphorylation,**

where **photophosphorylation* = Blue Light + Red Light,** so carbon fixation can be 0, 1, 2, since photophosphorylation can take the values 0, 1, and 2.

**MCPS^*^ = (blue light + red light) × (C_i_>0)**

Since the nodes CO_2_, blue light, red light have a fixed state in any simulated condition, photophosphorylation will stabilize as well. A necessary condition for carbon fixation or MCPS to oscillate is that C_i_ oscillates between 0 and positive values.

The C_i_ regulatory function can be represented with a truth table:

| **CO_2_** | **carbon fixation, MCPS** | **C_i_*** |
| --- | --- | --- |
| CO_2_=0 | Anything | 0 |
| CO_2_=1 | Max(carbon fixation, MCPS)=2 | 0.5 |
|  | Max(carbon fixation, MCPS)!=2 | 1 |
| CO_2_=2 | Anything | 2 |

Since the only way C_i_ can transit from 0 to a positive value is that CO_2_ changes value from 0 to 1 or 2, this necessary condition cannot be met in any of the conditions considered. Hence, there cannot be oscillation in the nodes carbon fixation and MCPS in the long term, as long as the regulatory function of C_i_ is not perturbed. This implies that the value of carbon fixation and MCPS stabilizes. This in turn implies that C_i_ cannot oscillate, because all of its inputs stabilize.

The perturbation scenarios we consider entail fixing the state of a node. Since no such perturbation changes the regulatory function of C_i_, there will not be an oscillation in this SCC. This conclusion is maintained in the reduced model since no node in this SCC is affected by the simplification.

1. **Simplification of the model**

We first show that the edge [malate^2-^]_a_→AnionCh is redundant in the long term. The AnionCh regulatory function in the Sun et al. model can be expressed by the following truth table:

| **Anion_highactivation_** | **PIP2_PM_** | **AnionCh^*^** |
| --- | --- | --- |
| 0 | 0 | 1 |
|  | 1 | 0 |
| 1 | 0 or 1 | 1.6 |

where

**Anion_highactivation_ = (([Ca^2+^]_c_ = 2) *Or* ABA) *And Not* ABI1 *Or* (C_i_ = 2) *Or* ([malate^2-^]_a_ = 2)**

We also need to consider the regulatory function of [malate^2-^]_a_:

**[malate^2-^]_a_^*^ = ((MCPS > 0) *Or* ([malate^2-^]_c_> 0) *And* (AnionCh > 0) *And* (PMV < 0)) × Max(1, C_i_)**

From the [malate^2-^]_a_ regulatory function we see that a necessary condition for [malate^2-^]_a_ to be 2 is C_i_=2. Note that C_i_ is proven to be stable in the long term, and in the AnionCh regulatory function C_i_ and [malate^2-^]_a_ are connected by “or”: “(C_i_=2) or ([malate^2-^]_a_=2)”. Hence, if C_i_ stabilizes at 2, AnionCh=1.6; if C_i_ is stabilized at some other value (i.e. 0 or 1), [malate^2-^]_a_ will not enter the value 2 in the long run. So “(C_i_=2) or ([malate^2-^]_a_=2)” is equivalent to “C_i_=2” in the long term behavior.

Thus the edge [malate^2-^]_a_→AnionCh is redundant and can be eliminated without affecting the attractors of the system. This edge is the only out-going edge from the group of nodes that represent anions, other than the edges that go to stomatal opening. When simplifying the regulatory function of stomatal opening, its dependence on anions is eliminated, making this group of nodes a sink group unrelated to stomatal opening, allowing their elimination from the network.

Next we show that the regulatory function for **[K^+^]_c_** can be simplified without any loss of information compared to the Sun et al. model. The original functions involved in the Sun et al. model are:

**[K^+^]_c_^*^ = H^+^ATPase_complex_ × [K^+^]_c regulation_ × Anion_regulation_**,

and **[K^+^]_c regulation_ = (K_in_ *Or* [K^+^]_v_ *And* KEV) *And* H^+^ATPase_complex_ *And Not* K_out_**

Plugging in the 2^nd^ equation into the 1^st^ we get：

**[K^+^]_c_*= [(K_in_ *Or* KEV *And* [K^+^]_v_) *And* *Not* K_out_] ×Anion_regulation_× H^+^ATPase_complex_**

Furthermore, **Anion_regulation_** is equivalent to **((H^+^ATPase_complex_ -AnionCh)>0)**. The regulatory functions for these components are:

**Anion_regulation_ = [NO_3_^-^]_c regulation_ *Or* [Cl^-^]_c regulation_ *Or* [malate^2-^]_c regulation_**

**[NO_3_^-^]_c regulation_ = [NO_3_^-^]_a_ *And* CHL1 *And* (H^+^ATPase_complex_ ≥ AnionCh)**

**[Cl^-^]_c regulation_ = (H^+^ATPase_complex_ ≥ AnionCh)**

**[malate^2-^]_c regulation_ = (*Not* (mito *And* ABA)) *And* (H^+^ATPAse_complex_ ≥ AnionCh)**

Since **(H^+^ATPase_complex_ ≥ AnionCh)** is a shared required (AND-connected) element of all three independent (OR-connected) clauses of **Anion_regulation,_** it is equivalent to **Anion_regulation_**. Therefore we can simplify the regulatory function of **[K^+^]_c_**:

**[K^+^]_c_*= [(K_in_ *Or* KEV *And* [K^+^]_v_) *And* *Not* K_out_] ×(H^+^ATPase_complex_ ≥ AnionCh) × H^+^ATPase_complex_**

Now that we have shown that the network simplification is valid, we can use the reduced model, which has only 32 nodes, for the remaining analysis.

1. **The NO cycle does not admit any oscillations or multi-stability under any perturbation**

The NO cycle is composed of the nodes PLD, ROS, NO, and the three positive edges between them. It does not have any inhibitory edges, so it cannot oscillate; it is a single cycle, so perturbation within the cycle will break the loop. Hence under no perturbation can this cycle oscillate.

We evaluate the possibility of multi-stability by looking at the regulatory functions of the three nodes:

**PLD *= ABA + NO**

**ROS *= (photophosphorylation>0) *And* (PLD>0) *And Not* ABI1**

**NO*= (photophosphorylation>0) *And* ROS**

We also need the nodes:

**ABI1 *= *Not* ABA**

**photophosphorylation *= Blue Light + Red Light**

If ABA=0, then ROS becomes 0, thus NO becomes 0, and thus PLD becomes 0; if ABA =1, then ABI =0 and PLD>0 which means ROS=NO=photophosphorylation, thus everything stabilizes at a value determined by Blue Light and Red Light. So there is no multi-stability. The above analysis applies for any perturbation outside the NO cycle; a perturbation inside the cycle will break the cycle, disabling multi-stability. So the conclusion is that the NO cycle does not have multi-stability under any perturbation.

1. **The Ion SCC does not admit any multi-stability under any perturbation; any oscillations are limited to the [Ca^2+^]_c_ –Ca^2+^ATPase^[[1]](#footnote-1)^ negative feedback loop.**

For ease of understanding we reproduce the Ion SCC and its sole successor, Stomatal Opening.


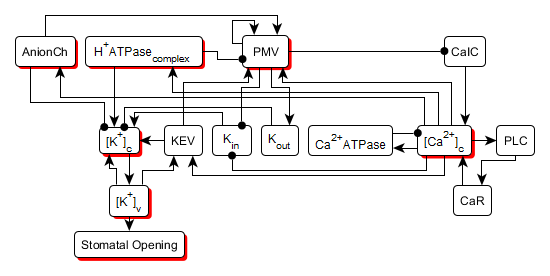


Figure S1. The Ion SCC with Stomatal Opening in the reduced model. Nodes with red shadows have multiple levels; the rest are Boolean. Potassium is the only ion present after network reduction. All regulators of this sub-network have been proven to be stable and are therefore omitted. The only successor of this SCC is Stomatal Opening (SO).

We prove that [Ca^2+^]_c_ oscillates in special way such that it will not affect most of its successors. Specifically, [Ca^2+^]_c_ cannot enter the state 2 in the long term.

[Ca^2+^]_c_ has regulators CaIC, CaR, PMV, and Ca^2+^ATPase. The relevant regulatory functions are:

**CaIC^*^ = ROS *And* (PMV ≤ 0)**

**CaR^*^ = NO *Or* PLC**

**PLC^*^ = Blue Light *Or* (ABA *And* ([Ca^2+^]_c_> 0))**

**NO = Nitrite *And* photophosphorylation *And* ROS**

**ROS^*^ = photophosphorylation *And* PLD *And not* ABI1**

**Ca^2+^ATPase^*^ = ([Ca^2+^]_c_> 0)**

**PMV^*^ = PMV – (H^+^ATPase_complex_>0) + ((AnionCh>0) *And* (PMV<0)) + (([Ca^2+^]_c_ = 2) *Or* KEV)**

**[Ca^2+^]_c_^*^ = ((CaIC *Or* CaR) *And Not* Ca^2+^ATPase) + ABA**

To help interpret the regulatory function of [Ca^2+^]_c_, we convert it into the following truth table:

| **CaIC or CaR** | **ABA** | **Ca^2+^ATPase** | **[Ca^2+^]_c_*** |
| --- | --- | --- | --- |
| CaIC or CaR =True | 0 | 1 | 0 |
|  | 0 | 0 | 1 |
|  | 1 | 0 | 2 |
|  | 1 | 1 | 1 |
| CaIC or CaR =False | 0 | Any | 0 |
|  | 1 |  | 1 |

[Ca^2+^]_c_ can have states 0,1, and 2.

First, [Ca^2+^]_c_ cannot be stable at 2. Let’s suppose [Ca^2+^]_c_=2 and is stable, then: [Ca^2+^]_c_=2 => Ca^2+^ATPase=1 => [Ca^2+^]_c_=1, which is a contradiction. Hence [Ca^2+^]_c_ cannot be stable at 2.

From the [Ca^2+^]_c_ truth table, we know that a necessary condition for [Ca^2+^]_c_ entering 2 is ABA=1. However under ABA=1, [Ca^2+^]_c_ cannot enter 0, which means that Ca^2+^ATPase=1, thus under this situation [Ca^2+^]_c_ can only stabilize at [Ca^2+^]_c_ = 1. Note that since this conclusion does not require any other condition than fixed inputs, [Ca^2+^]_c_ cannot enter 2 under any perturbation.

The regulatory functions of the rest of the network are dependent only on [Ca^2+^]_c_=2, that is, if [Ca^2+^]_c_!=2, [Ca^2+^]_c_ will not affect any other nodes. So given this conclusion, the sub-network composed of CaIC, [Ca^2+^]_c,_ Ca^2+^ATPase, PLC and CaR will not affect other parts of the network, i.e. it is a sink network component, and therefore can be ignored when analyzing other sub-networks. See Figure 3 in the main text for a graph of the simplified sub-network with edges reduced.

Next we analyze the long term behavior of the calcium sub-network. It has two cycles. The [Ca^2+^]_c_ - Ca^2+^ATPase cycle is a negative feedback loop. It cannot have multi-stability under any perturbation, but it can oscillate.

The [Ca^2+^]_c_ -PLC-CaR cycle is a positive feedback loop. It cannot oscillate under any perturbation. We analyze each regulatory function with a given ABA value. ABA=1 => [Ca^2+^]_c_ >0 => Ca^2+^ATPase=1 => [Ca^2+^]_c_ =1 => PLC=1, everything will stabilize. ABA=0 => PLC=Blue light, everything will stabilize. So there is no multi-stability under any perturbation. Thus we can conclude that the [Ca^2+^]_c_ sub-network does not have multi-stability under any perturbation; except for the [Ca^2+^]_c_, Ca^2+^ATPase cycle, the sub-network cannot oscillate under any perturbation.

Now we remove all edges that depend on ([Ca^2+^]_c_ = 2), and proceed to the remaining part of the ion SCC. AnionCh and H^+^-ATPase_complex_ are not a part of an SCC anymore. The node KEV has the regulatory function:

**KEV^*^ = [K^+^]_v_ *And* ([Ca^2+^]_c_ = 2)**

This means KEV must eventually stabilize at 0 since [Ca^2+^]_c_ cannot be 2 in the long term. Then we consider the regulatory function of PMV, which includes complex self-regulation:

**PMV* = PMV- bool(H^+^ATPase_complex_>0) + (AnionCh *And* (PMV<0)) + (([Ca^2+^]_c_ = 2) *Or* KEV)**

Since the edge KEV→ PMV is redundant in the long term, the PMV regulatory function is simplified to:

**PMV* = PMV- bool(H^+^ATPase_complex_>0) + (AnionCh *And* (PMV<0)),**

where both H^+^ ATPase_complex_ and AnionCh were shown to stabilize in the long term. PMV can have the states {-2,-1,0,1,2}. Other nodes only need the sign of PMV, i.e. only expressions of (PMV<0) and (PMV>0) are used in all other nodes’ regulatory functions. In this sense the states {-2,-1} of PMV are equivalent, and the states {1,2} are equivalent, too. Considering the possible values of bool(H^+^ATPase_complex_>0), if H^+^ATPase_complex_>0 then PMV will stabilize at a negative value, no matter what level AnionCh is. If H^+^ATPase_complex_=0, PMV will stabilize on a level within the set {0,1,2}, no matter what level AnionCh is, because AnionCh only affects PMV when PMV is negative. So there is no oscillation in this node under any perturbation.

Next we consider conditions for multi-stability of PMV. Recall that if H^+^ATPase_complex_ >0 is true in the long term, PMV will stabilize at a negative value, and it is not important which negative value. So there is no multi-stability when H^+^ATPase_complex_>0. For H^+^ATPase_complex_=0 cases multi-stability is possible, as PMV can be zero or positive. Since the regulatory function for [K^+^]_c_ is:

**[K^+^]_c_*= [(K_in_ *Or* KEV *And* [K^+^]_v_) *And Not* K_out_] ×(H^+^ATPase_complex_ ≥ AnionCh) × H^+^ATPase_complex,_**

in these cases [K^+^]_c_=0, which in turn means that the ion contribution to stomatal opening will be 0. So we have our first conclusion that in all possible cases of multi-stability in PMV, H^+^ATPase_complex_ is required to be zero, and the ion contribution to stomatal opening is zero. Since the multi-stability can only happen with non-negative values, only a single node, K_out_, is affected, as it is the sole node with (PMV>0) in its regulatory function:

**Kout* = (ABA *Or* (Ci=2) *Or* (*Not* ROS) *Or Not* NO *Or Not* FFA) *And* (PMV>0)**

Thus K_out_ can display multi-stability in response to PMV multi-stability. The only node K_out_ regulates is [K^+^]_c_, which stabilizes at 0 in multi-stability cases under any perturbation because H^+^ATPase_complex_ is zero. Thus the maximal difference between two attractors is in PMV and K_out_ and the attractors have the same level of stomatal opening (specifically, 0 or 1, see Table 3 in the main text). Note that although this conclusion is only true for perturbations of nodes other than [K^+^]_c_, it is practically infeasible to control [K^+^]_c_. Therefore we conclude that potential multi-stability of PMV has very limited effect and does not affect stomatal opening for all biologically meaningful situations. These conclusions are consistent with the simulation results from the stable motifs algorithm.

Now we proceed to the remaining part of the Ion SCC. The [K^+^]_c_ regulatory function has (KEV and [K^+^]_v_), when KEV is 0 in the long term, the edge [K^+^]_v_ → [K^+^]_c_ will also disappear. Then there are no more cycles left in this sub-network. Also, K_out_ does not affect any other node when it can have multi-stability. So the conclusion is that the rest of the sub-network cannot oscillate or have multi-stability under any perturbation. Thus, there is no oscillation within the Ion SCC, except in the [Ca^2+^]_c_ -Ca^2+^ATPase cycle, under any perturbation. There is no multi-stability within the Ion SCC, except in the node PMV, under any perturbation.

**Conclusion:** In summary, we have shown that in the reduced model any oscillations are limited to the [Ca^2+^]_c_ –Ca^2+^ATPase negative feedback loop, and any multi-stability is limited to the PMV, K_out_ nodes, under any perturbation.

1. **Possibility of multi-stability and oscillations in the original Sun et al. model**

To complete our analysis, now we consider the Sun. et al model before reduction is applied. As we have shown in section 1, the C_i_ SCC does not admit oscillations or multi-stability under any perturbation. The original NO cycle does not admit oscillations or multi-stability under any perturbation either, because in the reduction only simple mediator nodes are removed, which does not change the attractors.

For the same reason, our conclusions on the simplified Ion SCC, specifically on the [Ca^2+^]_c_ –Ca^2+^ATPase oscillation and PMV-K_out_ multi-stability, hold for the original model. Now we need to consider the 10 anion-related nodes ([malate^2-^]_a_, [malate^2-^]_c_, starch, [Cl^-^]_c_, [NO_3_^-^]_c_, [NO_3_^-^]_a_, ROP2, RIC7, ABC, and PEPC), which were ignored in the simplification of the Ion SCC. First, notice that we can use the conclusion that [Ca^2+^]_c_ –Ca^2+^ATPase cannot enter level 2, as the derivation of that statement has no requirement on these anion nodes. Also, the anion nodes are regulated by (PMV>0), thus they are not affected by PMV multi-stability. We can exploit these properties and reduce the Ion SCC to to 12 nodes, as shown in Figure S2.


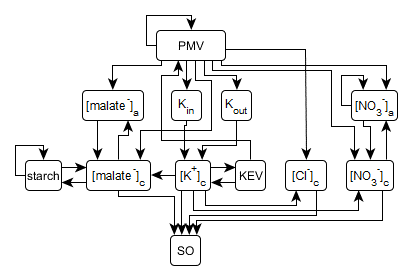


Figure S2. The Ion SCC in the Sun et al. model [[5](#_ENREF_5)], after taking into account that [Ca^2+^]_c_ cannot equal 2 and reducing related nodes and edges. All stable predecessors are omitted, because they do not induce multi-stability or oscillation. For simplicity we are not showing red shadows here, because we only need to find the SCCs.

There are two SCCs, one formed by [NO_3_­^-^]_a_ and [NO_3_­^-^]_c_, and the other is formed by starch – [malate^-^]_c_ – [malate^-^]_a_. There are no negative feedback loops, so there cannot be oscillations under any perturbation.

The NO­_3_^-^ two node SCC has node functions:

**[NO_3_^-^]_a_^*^ = [NO_3_^-^]_a_ *Or* [NO_3_^-^]_c_ *And* AnionCh *And* (PMV < 0)**

**[NO_3_^-^]_c_^*^ = [K^+^]_c regulation_ × [NO_3_^-^]_c regulation_**

**[NO_3_^-^]_c regulation_ = [NO_3_^-^]_a_ *And* CHL1 *And* (H^+^ATPase_complex_ ≥ AnionCh)**

[NO_3_^-^]_a_ is assumed to not be a limiting factor in the Sun et al model; therefore, it is initialized at level 1 (ON) and according to its function it will remain ON. This means that there is no multi-stability in this SCC, as [NO_3_^-^]_c_ cannot have multiple stable values when none of its inputs have multiple stable values.

The malate^2-^ three node SCC has node functions:

**[malate^2-^]_a_^*^ = ((MCPS > 0) *Or* ([malate^2-^]_c_ > 0) *And* (AnionCh > 0) *And* (PMV < 0)) × Max{1, C_i_}**

**starch^*^ = starch *Or* ([malate^2-^]_c_ > 0) *And* ABA**

**[malate^2-^]_c_^*^ = 0.5 × (internal + import) × [K^+^]_c regulation_ × [malate^2-^]_c regulation,_**

with intermediate values given as:

**[malate^2-^]_c regulation_ = (*Not* (mitochondria *And* ABA)) *And* (H^+^ATPase_complex_ ≥ AnionCh)**

**internal = (starch *Or* carbfix)×PEPC**

**import = [malate^2-^]_a_ *And* AtABCB14**

**[K^+^]_c regulation_ = (K_in_ *Or* [K^+^]_v_ *And* KEV) *And* H^+^-ATPAse_complex_ *And Not* K_out_**

Starch is assumed to not be a limiting factor in the Sun et al. model. The state of starch is assumed to be ON initially, and therefore will remain ON because of its rule. Under this condition, internal = PEPC = *Not* ABA. In addition, AtABCB14 = [malate^2-^]_a_; and mitochondria is a source node assumed to be ON by Sun et al. Then we can write a simplified function for [malate^2-^]_c_:

**[malate^2-^]_c_^*^ = 0.5 × {(*Not* ABA) + [malate^2-^]_a_ } × {(*Not* ABA) *And* (H^+^ATPase_complex_ ≥ AnionCh)}**

If ABA = 1, then this function is 0; if ABA = 0, the sign of the function is independent of [malate^2-^]_a_, which can take the values 0,1,2. Here we care about the sign of [malate^2-^]_c_ because[malate^2-^]_a_ only depends of the sign of [malate^2-^]_c_. Since the sign is independent of [malate^2-^]_a_, we know this loop is non-functional, i.e. varying [malate^2-^]_a_ will not feedback to itself. This means that there cannot be multi-stability, given that starch is initially ON.

To conclude, we know that given the initial conditions [NO_3_^-^]_a_ = ON and starch = ON assumed in the Sun et al. model, the reduced anion nodes cannot have multi-stability.

The original Sun et al. model contains an additional negative feedback loop between RIC7 and stomatal opening. This part is reduced in the reduced model. The regulatory functions are:

**RIC7 = ROP2 *×* (SO>=7)**

**SO = a stable value – (RIC7)/6**

Both ROP2 and RIC7 are Boolean variables. ROP2 stabilizes for any input combination. As it is a negative feedback loop, it cannot have multi-stability but may oscillate. There can indeed be oscillation within this part, specifically, a low amplitude (less than 0.17) oscillation around SO=7. Such oscillation can be observed under some special perturbations. For example, when the input signals are ABA=0, CO_2_=C_i_=1, Blue Light =1, Red Light=1, and perturbations ROS=1 and sucrose =0 are applied, oscillation of SO between 7.09 and 6.92, with RIC7 oscillating between 0 and 1, can be observed. There are also perturbation settings that result in SO=6.9 or SO=7.008, which are very close to causing an oscillation. However this oscillation depends on the parameters of the model, for example the assumed threshold SO>=7, and the denominator 6. The high dependence on parameters, together with the low amplitude of the oscillation, suggest that this oscillation within the RIC7 path may have low biological relevance.

To conclude this section, we state that given the initial conditions [NO_3_^-^]_a_ = ON and starch = ON assumed in the Sun et al. model, the reduced anion nodes cannot have multi-stability. As for oscillation, the RIC7 path in the stomatal opening model is capable of oscillating, but the oscillation has a low biological relevance.

**References**

1. Richard, A. and J.-P. Comet, *Necessary conditions for multistationarity in discrete dynamical systems.* Discrete Applied Mathematics, 2007. **155**(18): p. 2403-2413.

2. Richard, A., *Negative circuits and sustained oscillations in asynchronous automata networks.* Advances in Applied Mathematics, 2010. **44**(4): p. 378-392.

3. Remy, E., P. Ruet, and D. Thieffry, *Graphic requirements for multistability and attractive cycles in a Boolean dynamical framework.* Advances in Applied Mathematics, 2008. **41**(3): p. 335-350.

4. Thomas, R. and European Molecular Biology Organization., *Kinetic logic : a Boolean approach to the analysis of complex regulatory systems : proceedings of the EMBO course "Formal analysis of genetic regulation," held in Brussels, September 6-16, 1977*. Lecture notes in biomathematics. 1979, Berlin ; New York: Springer-Verlag. xiii, 507 p.

5. Sun, Z., et al., *Multi-level modeling of light-induced stomatal opening offers new insights into its regulation by drought.* PLoS Comput Biol, 2014. **10**(11): p. e1003930.

1. To distinguish from the subtraction operator ‘–‘, all dashes in the node names of this file are removed. Ca^2+^-ATPase is written as Ca^2+^ATPase, and H^+^ -ATPase_complex_ is written as H^+^ ATPase_complex_ [↑](#footnote-ref-1)
