# Supplementary material for: Analysis of a dynamic model of guard cell signaling reveals the stability of signal propagation
Source: BMC Syst Biol. 2016 Aug 19;10:78. doi: 10.1186/s12918-016-0327-7 (PMC4992220; doi:10.1186/s12918-016-0327-7)
Supplement: Additional file 4: — Examples of converting a multi-level update function to Boolean. (DOCX 38 kb) [file 12918_2016_327_MOESM4_ESM.docx]

Examples of converting a multi-level regulatory function into a Boolean regulatory function

In general we code the Boolean nodes with binary representation of integer numbers.

| **Multi-level node** | **Bool. Node_1** | **Bool. Node_2** |
| --- | --- | --- |
| 0 | 0 | 0 |
| 1 | 0 | 1 |
| 2 | 1 | 0 |
| 3 | 1 | 1 |
| … | Add more nodes | |

Sometimes the nodes can be better interpreted using a slightly different binary representation. For example, PLD is a node that can take states {0, 1, 2}. Its conversion mapping is

| **Multi-level PLD** | **PLD_high** | **PLD** |
| --- | --- | --- |
| 0 | 0 | 0 |
| 1 | 0 | 1 |
| 2 | 1 | 1 |

where the Boolean variable ‘PLD’ means the presence of PLD, and the Boolean variable ‘PLD_high’ means the high concentration of PLD. Thus we code the state 2 with the combination of Boolean variables “11” (meaning both presence and high level) instead of “10”, because it is a more natural reflection of a higher level of PLD. This is one of the examples where a converted Boolean node can have an explicit meaning. For multi-level nodes with many states, this technique is likely to fail. In those cases, we use binary coding without associating a specific meaning for each node. To complete the PLD example, we provide its regulatory functions before and after conversion:

Multi-level version:

**PLD *= ABA + NO** (Addition of two Boolean inputs, yields 2 when both inputs are true.)

Boolean version:

**PLD *= ABA *Or* NO**

**PLD_high *= ABA *And* NO**

Next we demonstrate how to convert a multi-level regulatory function with many states to a set of Boolean regulatory functions through some examples:

**1. Regulatory function of PP1­_cc_:**

In the reduced model, PP1_cc_ is a function of Blue Light, phot1complex, and PLD. Blue Light and phot1complex are Boolean, PLD has states {0, 1, 2}, PP1_cc_ can have states: {1, 1.5, 2, 3, 3.5, 4}.

The regulatory function of PP1_cc_ can be represented by a truth table as following:

| **Blue Light** | **phot1_complex_** | **PLD** | **PP1_cc_^*^** |
| --- | --- | --- | --- |
|  |  |  |  |
| 0 | 0 | 0 | 2 |
|  |  | 1 | 1.5 |
|  |  | 2 | 1 |
|  | 1 | 0 | 4 |
|  |  | 1 | 3.5 |
|  |  | 2 | 3 |
| 1 | any | 0 | 4 |
|  |  | 1 | 3.5 |
|  |  | 2 | 3 |

The multi-level PP1_cc_ is represented by three nodes, ‘PP1cc_1’, ‘PP1cc_2’ and ‘PP1cc_3’, coded with the table below:

| **Multi-level PP1_cc_** | **PP1cc_1** | **PP1cc_2** | **PP1cc_3** |
| --- | --- | --- | --- |
| 0 | 0 | 0 | 0 |
| 1 | 0 | 0 | 1 |
| 1.5 | 0 | 1 | 0 |
| 2 | 0 | 1 | 1 |
| 3 | 1 | 0 | 0 |
| 3.5 | 1 | 0 | 1 |
| 4 | 1 | 1 | 0 |

So the truth table becomes the following. Note that the state of PLD is now represented by two Boolean nodes, PLD_high and PLD.

| **Blue Light** | **phot1_complex_** | **PLD_high** | **PLD** | **PP1cc_1*** | **PP1cc_2*** | **PP1cc_3*** |
| --- | --- | --- | --- | --- | --- | --- |
| 0 | 0 | 0 | 0 | 0 | 1 | 1 |
|  |  | 0 | 1 | 0 | 1 | 0 |
|  |  | 1 | 1 | 0 | 0 | 1 |
|  | 1 | 0 | 0 | 1 | 1 | 0 |
|  |  | 0 | 1 | 1 | 0 | 1 |
|  |  | 1 | 1 | 1 | 0 | 0 |
| 1 | any | 0 | 0 | 1 | 1 | 0 |
|  |  | 0 | 1 | 1 | 0 | 1 |
|  |  | 1 | 1 | 1 | 0 | 0 |

To construct the regulatory function of each Boolean node, we list all combinations of input combinations where a Boolean node is 1, and connect them with the “Or” rule. For example, PP1cc_1 is 1 when (Blue light=0 and phot1_complex_=1), or when (Blue light=1), so the regulatory function for PP1cc_1 is

**PP1cc_1 *= (*Not* Blue Light *And* phot1_complex_) *Or* Blue Light,**

which can be simplified to

**PP1cc_1 *= Blue Light *Or* phot1_complex_.**

The same is done for PP1cc_2 and PP1cc_3:

**PP1cc_2 *= ((phot1_complex_ *Or* Blue Light) *And Not* PLD *And Not* PLD_high) *Or* (*Not* (phot1_complex_ *Or* Blue Light) *And Not* PLD_high)**

**PP1cc_3 *= (*Not* (phot1_complex_ *Or* Blue Light) *And Not* (PLD *And Not* PLD_high)) *Or* ((phot1_complex_ *Or* Blue Light) *And* PLD *And Not* PLD_high)**

The stable motif algorithm does not require that the regulatory functions are expressed in the simplest way, so we did not attempt to simplify every regulatory function.

**2. Regulatory functions of PMV:**

We simplify the regulatory function of the multi-leveled node PMV, and use only two Boolean nodes, each with clear biological meaning, to represent it. The multi-level PMV regulatory function is:

**PMV* = PMV- bool(H^+^ATPase_complex_>0) + (AnionCh *And* (PMV <0)) + (([Ca^2+^]_c_ = 2) *Or* KEV)**

Here PMV can have states -2, -1, 0, 1, 2. We group the two positive values and the two negative values together as a simplification, and represent PMV with two Boolean nodes, PMV_neg and PMV_pos. We can apply this simplification because for all nodes regulated by PMV, other than itself, only the sign of PMV matters (i.e. only (PMV<0) and (PMV>0) appeared in other functions). This is because the distinction between hyperpolarization (PMV<0) and depolarization (PMV>0) of the plasma membrane is the most meaningful biologically. The converted Boolean regulatory functions of PMV are provided in Additional File 5.

**3. Regulatory functions of [K^+^]_c_, [K^+^]_v_ , H^+^ATPase_complex_, and SO.**

As shown in S3, the regulatory function of Stomatal Opening depends on [K^+^]_v_ and sucrose:

| **[K^+^]_v_** | **Sucrose** | **SO^*^** |
| --- | --- | --- |
| 0 | 0 | 0 |
|  | Sucrose >0 | 1 |
| 1<=[K^+^]_v_ <2 | Any | 2 |
| 2<=[K^+^]_v_ <6 | Any | 3 |
| 6<=[K^+^]_v_<9 | Any | 5 |
| 9<=[K^+^]_v_ | Any | 6 |

The regulatory functions for [K^+^]_c_ and [K^+^]_v_ are:

**[K^+^]_c_* = [(Kin or KEV and [K^+^]_v_) and not Kout] × ((H^+^ATPase_complex_ -AnionCh)>0) ×H^+^ ATPase_complex_**

**[K^+^]_v_* = [K^+^]_c_**

We had previously decided to not simplify [K^+^]_v_ in order to avoid a self-loop on [K^+^]_c_, which may cause interpretation difficulties. But encoding both [K^+^]_c_ and [K^+^]_v_ with 3 Boolean nodes each would cause unnecessary complexity. Thus we perform a transformation that leaves the SO regulatory function unchanged (except for a time delay) but makes [K^+^]_c_ and [K^+^]_v_ Boolean variables.

The regulatory function of the Boolean [K^+^]_c_ is the Boolean part of the original [K^+^]_c_ regulatory function, i.e.

**[K^+^]_c_* = [(Kin or KEV and [K^+^]_v_) and not Kout] × ((H^+^ATPase_complex_ -AnionCh)>0)**

The Boolean [K^+^]_v_ is still [K^+^]_v_* = [K^+^]_c_ , and in the SO table ‘[K^+^]_v_’ is replaced by ‘[K^+^]_v_ × H^+^ATPase_complex_’.

This action simplifies [K^+^]_c_ and [K^+^]_v_ to Boolean variables, without causing any meaningful change to the dynamics of the system, as [K^+^]_c_ and [K^+^]_v_ do not affect other nodes with their multi-level states.

H^+^ATPase_complex_ is mapped into 3 Boolean nodes with the table below:

| H^+^ATPase_complex_  (Original) | HATPase_1 | HATPase_2 | HATPase_3 |
| --- | --- | --- | --- |
| 0 | 0 | 0 | 0 |
| 0.5 | 0 | 0 | 1 |
| 0.9 | 0 | 0 | 1 |
| 1 | 0 | 1 | 0 |
| 1.5 | 0 | 1 | 1 |
| 1.8 | 1 | 0 | 0 |
| 2 | 1 | 0 | 1 |
| 2.7 | 1 | 0 | 1 |
| 3 | 1 | 0 | 1 |
| 4.5 | 1 | 0 | 1 |
| 6 | 1 | 1 | 0 |
| 9 | 1 | 1 | 1 |

Since SO is primarily decided by ‘[K^+^]_v_ × H^+^ATPase_complex_’, its level is the same as H^+^ATPase_complex_ if [K^+^]_v_ is ON. Then it is convenient to define three Boolean variables for stomatal opening, SO_i = [K^+^]_v_ and H^+^ATPase_i, i=1,2,3. We represent the outcome of the situation with [K+]_v_=0 and nonzero sucrose as 010, which yields

**SO_1* = HATPase_1 *And* [K^+^]_v_**

**SO_2* = HATPase_2 *And* [K^+^]_v_ *Or* (*Not* [K^+^]_v_ *And* sucrose)**

**SO_3* = HATPase_3 *And* [K^+^]_v_**

The overall mapping from the Boolean SO nodes to the simplified multi-level SO is summarized in the following table:

| SO_1 | SO_2 | SO_3 | Simplified SO |
| --- | --- | --- | --- |
| 0 | 0 | 0 | 0 |
| 0 | 0 | 1 | 1 |
| 0 | 1 | 0 | 1 |
| 0 | 1 | 1 | 2 |
| 1 | 0 | 0 | 2 |
| 1 | 0 | 1 | 3 |
| 1 | 1 | 0 | 5 |
| 1 | 1 | 1 | 6 |

The list of the Boolean regulatory functions of all the nodes is provided in Additional File 5.
