# Supplementary material for: Analysis of a dynamic model of guard cell signaling reveals the stability of signal propagation
Source: BMC Syst Biol. 2016 Aug 19;10:78. doi: 10.1186/s12918-016-0327-7 (PMC4992220; doi:10.1186/s12918-016-0327-7)
Supplement: Additional file 5: — Regulatory functions of the Boolean-converted reduced stomatal opening model. (DOCX 25 kb) [file 12918_2016_327_MOESM5_ESM.docx]

Regulatory Functions of the Boolean- Converted Reduced Stomatal Opening Model

Here we provide the Boolean-converted reduced model’s regulatory functions as used in the stable motif algorithm. Several node names have been modified to satisfy the format requirements of the algorithm (e.g. no subscripts or superscripts are allowed). We provide a list of node names used here compared with their names used previously:

| **Node name in this file** | **Node name previously** |
| --- | --- |
| BL | Blue Light |
| RL | Red Light |
| ABA | ABA |
| CO2 | CO_2_ |
| CO2_high |  |
| phot1_complex | Phot1­_complex_ |
| PLC | PLC |
| PLA2 | PLA_2_β |
| CaIC | CaIC |
| CaR | CaR |
| NO | NO |
| CaATPase | Ca^2+^ATPase |
| FFA | FFA |
| Kin | K_in_ |
| Kout | K_out_ |
| KEV | KEV |
| Sucrose | Sucrose |
| Phph | Photophosphorylation |
| Phph_high |  |
| Carbfix | Carbon fixation |
| Carbfix_high |  |
| PLD | PLD |
| PLD_high |  |
| ABI1 | ABI1 |
| ROS | ROS |
| MCPS | MCPS |
| MCPS_high |  |
| Kc | [K^+^]_c_ |
| Kv | [K^+^]_v_ |
| AnionCh | AnionCh |
| AnionCh_high |  |
| Ci | C_i_ |
| Ci_sup |  |
| PMV_neg | PMV |
| PMV_pos |  |
| PP1cc_2 | PP1_cc_ |
| PP1cc_2 |  |
| PP1cc_3 |  |
| PK_1 | Protein kinase |
| PK_2 |  |
| PK_3 |  |
| HATPase_1 | H^+^ATPase_complex_ |
| HATPase_2 |  |
| HATPase_3 |  |
| Cac | [Ca^2+^]_c_ |
| Cac_high |  |
| SO_1 | Stomatal opening |
| SO_2 |  |
| SO_3 |  |

The following are the regulatory functions put in the format required by the stable motif algorithm. Additional File 6 is the .txt file with all regulatory functions of the Boolean-converted reduced model ready to be run by the stable motif algorithm.

The stable motif algorithm and instructions on how to use it can be found from this link: https://github.com/jgtz/StableMotifs

*Input signals: (one can set these to the needed initial values)*

BL* = 1

RL* = 1

ABA* = 1

CO2* = 1

CO2_high* = 0

phot1_complex* = BL

PLC* = BL or ABA and Cac

PLA2* = phot1_complex or BL or RL

CaIC* = ROS and PMV_neg

CaR* = NO or PLC

NO* = phph and ROS

CaATPase* = Cac

FFA* = PLA2

Kin* = (FFA or not Cac_high or ABA) and (not (Ci and Ci_sup))and PMV_neg

Kout* = (ABA or (Ci and Ci_sup) or (not ROS) or not NO or not FFA) and PMV_pos

KEV* = Cac_high and (Kv)

sucrose* = carbfix and not ABA

phph* = BL or RL

phph_high* = BL and RL

carbfix* = (CO2 or Ci) and phph

carbfix_high* = (CO2 or Ci) and phph_high

PLD* = ABA or NO

PLD_high* = ABA and NO

ABI1* = not ABA

ROS* = phph and (PLD and not ABI1)

MCPS* = (BL or RL) and (Ci or Ci_sup)

MCPS_high* = (BL and RL) and (Ci or Ci_sup)

Kc* = ((Kin or KEV and (Kv)) and not Kout) and (HATPase_1 or (HATPase_2 and HATPase_3 and not AnionCh_high) or (HATPase_2 or HATPase_3) and (not AnionCh and not AnionCh_high)) Kv* = Kc

AnionCh* = ((((Cac_high) or ABA) and not ABI1) or (Ci and Ci_sup)) or (not ((((Cac_high) or ABA) and not ABI1) or (Ci and Ci_sup)) and not (phot1_complex or BL))

AnionCh_high* = (((Cac_high) or ABA) and not ABI1) or (Ci and Ci_sup)

Ci* = not((not CO2) or (CO2 and not CO2_high and (carbfix_high or MCPS_high)))

Ci_sup* = not((not CO2) or (CO2 and not CO2_high and not (carbfix_high or MCPS_high)))

PMV_neg *= ((HATPase_1 or HATPase_2 or HATPase_3) and (((AnionCh and PMV_neg) and not (Cac_high or KEV)) or (not (AnionCh and PMV_neg) and ((PMV_neg) or ((not PMV_pos and not PMV_neg) and not (Cac_high or KEV)))))) or (not (HATPase_1 or HATPase_2 or HATPase_3) and (not (AnionCh and PMV_neg))and PMV_neg and not (Cac_high or KEV))

PMV_pos *= ( not (HATPase_1 or HATPase_2 or HATPase_3) and (((AnionCh and PMV_neg) and (Cac_high or KEV)) or (not (AnionCh and PMV_neg) and not PMV_neg and PMV_pos) or (not (AnionCh and PMV_neg) and (not PMV_pos and not PMV_neg) and (Cac_high or KEV)))) or ((HATPase_1 or HATPase_2 or HATPase_3) and (not (AnionCh and PMV_neg)) and not PMV_neg and PMV_pos and (Cac_high or KEV))

PP1cc_1* = phot1_complex or BL

PP1cc_2* = ((phot1_complex or BL)and not PLD and not PLD_high) or (not (phot1_complex or BL)and not PLD_high)

PP1cc_3* = (not (phot1_complex or BL)and not (PLD and not PLD_high)) or ((phot1_complex or BL)and PLD and not PLD_high)

PK_1* = ((not Ci and not Ci_sup) and ((PP1cc_1 and PP1cc_2 and not PP1cc_3) or (not PP1cc_1 and PP1cc_2 and PP1cc_3) or (PP1cc_1 and not PP1cc_2))) or ((not Ci and Ci_sup) and ((PP1cc_1 and not PP1cc_2 and PP1cc_3) or (PP1cc_1 and PP1cc_2 and not PP1cc_3))) or ((Ci and not Ci_sup) and (PP1cc_1 and PP1cc_2 and not PP1cc_3))

PK_2* = ((not Ci and not Ci_sup) and not ((not PP1cc_1 and PP1cc_2 and PP1cc_3)or (PP1cc_1 and not PP1cc_2))) or ((not Ci and Ci_sup) and ((PP1cc_1 and not PP1cc_3) or ( not PP1cc_1 and PP1cc_2 and PP1cc_3))) or ((Ci and not Ci_sup) and (PP1cc_1 and not PP1cc_2))

PK_3* = ((not Ci and not Ci_sup) and (PP1cc_1 and PP1cc_2 and not PP1cc_3)) or ((not Ci and Ci_sup) and ( PP1cc_1 and not PP1cc_2 and PP1cc_3)) or ((Ci and not Ci_sup) and ( PP1cc_1 and not PP1cc_2 and PP1cc_3))

HATPase_1* = ((FFA or PLA2) and not Cac_high and (PK_1 or PK_2 or PK_3)) and (((not PK_1 and PK_2 and PK_3) and phph) or (PK_1 and ((not PK_2 and not PK_3 and phph) or (not PK_2 and PK_3)or PK_2)))

HATPase_2* = ((FFA or PLA2) and not Cac_high and (PK_1 or PK_2 or PK_3)) and ((not PK_1 and (PK_2 and not PK_3 and phph)) or (PK_1 and ((not PK_2 and not PK_3 and not phph) or (PK_2 and not PK_3 and phph_high) or (PK_2 and PK_3 and phph))))

HATPase_3* = ((FFA or PLA2) and not Cac_high and (PK_1 or PK_2 or PK_3)) and ((not PK_1 and ((PK_2 and not PK_3 and (not phph or phph_high)) or (PK_2 and PK_3 and (not phph or phph_high)))) or (PK_1 and ((not PK_2 and not PK_3 and phph) or (not PK_2 and PK_3) or (PK_2 and not PK_3 and not phph_high) or (PK_2 and PK_3 and (not phph or phph_high)))))

Cac* = ((CaIC or CaR) and not CaATPase) or ABA

Cac_high* = ((CaIC or CaR) and not CaATPase) and ABA

SO_1* = HATPase_1 and Kv

SO_2* = HATPase_2 and Kv or (not Kv and sucrose)

SO_3* = HATPase_3 and Kv
