# Supplementary material for: Analysis of a dynamic model of guard cell signaling reveals the stability of signal propagation
Source: BMC Syst Biol. 2016 Aug 19;10:78. doi: 10.1186/s12918-016-0327-7 (PMC4992220; doi:10.1186/s12918-016-0327-7)
Supplement: Additional file 7: — GINsim attractor analysis of the Boolean-converted reduced stomatal opening model. (DOCX 42 kb) [file 12918_2016_327_MOESM7_ESM.docx]

**GINsim attractor analysis of the Boolean-converted reduced stomatal opening model**

The Boolean-converted reduced stomatal opening model, provided in in SBML format in Additional File 8, can be imported to GINsim to reproduce this analysis. GINsim and its tutorial are available at www.ginsim.org

1. **Stable steady state computation**

We use the “stable state computation” tool of GINsim and find 30 stable steady states (fixed points), summarized in Table S1. These agree with the stable steady states found by the stable motif algorithm, summarized in Table 4 in the main text.

**Table S1.** Summary of the stable steady states found by GINsim

| **BL** | **RL** | **CO_2_** | **CO_2__high** | **ABA** | **SO (Bool)** | **SO** | **PMV_pos bistability** |
| --- | --- | --- | --- | --- | --- | --- | --- |
| 0 | 0 | Any | Any | Any | 000 | 0 | Yes |
| 0 | 1 | 0 | 0 | 1 | 000 | 0 | No |
| 0 | 1 | 1 | Any | 1 | 000 | 0 | Yes |
| 1 | Any | 1 | 0 | 1 | 000 | 0 | No |
| 1 | Any | 1 | 1 | 1 | 000 | 0 | Yes |
| 0 | 1 | 1 | Any | 0 | 010 | 1 | Yes |
| 0 | 1 | 0 | 0 | 0 | 101 | 3 | No |
| 1 | Any | 0 | 0 | 1 | 101 | 3 | No |

The first 5 columns indicate the input signal combination. The setting CO_2__high=1 and CO_2_=0 is not included because it is not biologically meaningful. The “SO (Bool)” column indicates the state of the Boolean node combination representing stomatal opening. The “SO” column is the state of stomatal opening when converted back to an integer. The last column indicates whether bistability of PMV_pos can be observed under this setting. In those cases, two stable steady states with (PMV_pos=0, K_out_=0) and (PMV_pos=1, K_out_=1) can be observed. The rest of the nodes are unaffected by this two-node bistability.

1. **Verifying complex attractors**

We use the simulation function of GINsim to verify the complex attractors we found by stable motif analysis. We set the initial state of the simulation to be the partial steady state found by the stable motif algorithm, i.e. setting all stabilized nodes to be in their stabilized state, and leaving unstabilized nodes (namely, Cac and CaATPase) unspecified. Cac_high stabilizes at 0 in all the cases. With this setting, GINsim will map the entire state space of the system under the above constraint and identify the allowed state transitions. If the stabilized nodes indicated by stable motif analysis are correct, their state will be unchanged, and only two nodes will change state, Cac and CaATPase, thus the relevant state space has only 4 states.

Here we provide one example GINsim simulation. The initial state, including the input signals, is:

| Node | State | Node | State | Node | State |
| --- | --- | --- | --- | --- | --- |
| BL | 1 | ROS | 0 | MCPS_high | 0 |
| RL | 0 | PMV_neg | 1 | AnionCh | 0 |
| CO2 | 1 | CaATPase | * | AnionCh_high | 0 |
| CO2_high | 0 | FFA | 1 | PP1cc_1 | 1 |
| ABA | 0 | Kin | 1 | PP1cc_2 | 1 |
| Phot1_complex | 1 | Cac_high | 0 | PP1cc_3 | 0 |
| Phph | 1 | Ci | 1 | PK_1 | 1 |
| Phph_high | 0 | Ci_sup | 0 | PK_2 | 0 |
| PLD | 0 | Kout | 0 | PK_3 | 0 |
| NO | 0 | PMV_pos | 0 | HATPase_1 | 1 |
| PLD_high | 0 | KEV | 0 | HATPase_2 | 0 |
| PLC | 1 | Sucrose | 1 | HATPase_3 | 1 |
| Cac | * | Carbfix | 1 | Kc | 1 |
| PLA2 | 1 | Carbfix_high | 0 | SO_1 | 1 |
| CaR | 1 | ABI1 | 1 | SO_2 | 0 |
| CaIC | 0 | MCPS | 1 | SO_3 | 1 |

A ‘*’ means that the node state is unspecified, and GINsim will consider all states (two states, in this case). Note that although the state of Cac, the first Boolean node encoding [Ca^2+^]_c_, is unspecified, the state of the other Boolean node, Cac_high, is fixed. We will use the following representation to represent this state more efficiently:

| 1 | 0 | 1 | 0 | 0 | 1 | 1 | 0 | 0 | 0 | 0 | 1 | * | 1 | 1 | 0 | 0 | 1 | * | 1 | 1 | 0 | 1 | 0 | 0 | 0 | 0 | 1 | 1 | 1 | 0 | 1 | 1 | 0 | 0 | 0 | 1 | 1 | 0 | 1 | 0 |  | 0 | 1 | 0 | 1 | 1 | 1 | 0 | 1 |
| --- | --- | --- | --- | --- | --- | --- | --- | --- | --- | --- | --- | --- | --- | --- | --- | --- | --- | --- | --- | --- | --- | --- | --- | --- | --- | --- | --- | --- | --- | --- | --- | --- | --- | --- | --- | --- | --- | --- | --- | --- | --- | --- | --- | --- | --- | --- | --- | --- | --- |

The sequence follows the first column of the table from top to bottom, then the second column from top to bottom, then the third from top to bottom, e.g. the first five digits represent BL, RL, CO2, CO2_high, ABA, respectively. The 13^th^ digit and the 19^th^ digit represent Cac and CaATPase, respectively.

The GINsim simulation result is the graph of allowed state transitions (called state transition graph), as shown in Figure S3.


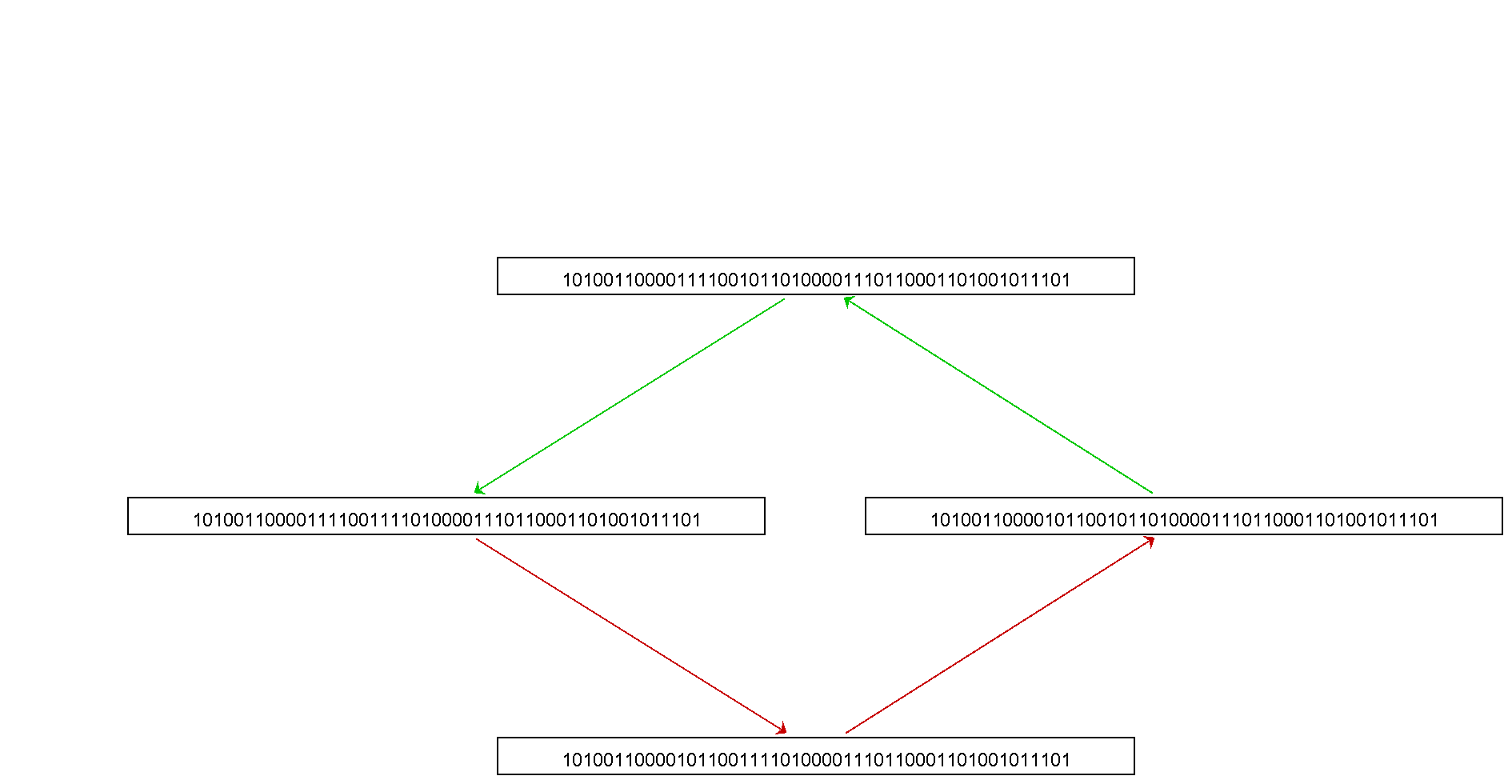


Figure S3. The state transition graph indicated by GINsim. Each node represents a state. A green edge indicates an increase in a node state during a state transition; a red edge indicates a decrease in a node state during a state transition. The 13^th^ digit and the 19^th^ digit represent Cac and CaATPase, respectively. They change alternatively between 1 and 0, forming an oscillation. The rest of the nodes remain in the same state.

This result confirms that the partial steady state found by stable motif analysis is indeed a partial steady state, because no additional state can be reached in state transitions.

The other 7 complex attractors are verified in the same way. For input combinations where bistability is present, each partial steady state is considered as a separate initial condition. The simulation results show that for all initial conditions the state transition graph is always a four-state strongly-connected component, with Cacand CaATPase being the nodes that oscillate.

Thus the GINsim simulation confirmed the complex attractors conclusions found by the stable motif algorithm. Moreover, the state transitions between the four states form a simple two node negative feedback oscillation, which is consistent with the theoretical analysis result.
